# Versatile properties of *Opuntia ficus-indica* (L.) Mill. flowers: *In vitro* exploration of antioxidant, antimicrobial, and anticancer activities, network pharmacology analysis, and *In-silico* molecular docking simulation

**DOI:** 10.1371/journal.pone.0313064

**Published:** 2024-11-04

**Authors:** Mai Ali Mwaheb, Nashwa Mohamed Reda, Mohammad S. El-Wetidy, Asmaa H. Sheded, Fatimah Al-Otibi, Gadah A. Al-Hamoud, Mohamed A. Said, Esraa A. Aidy

**Affiliations:** 1 Botany Department, Faculty of Science, Fayoum University, Fayoum, Egypt; 2 Clinical and Chemical Pathology Department, Faculty of Medicine, Cairo University, Cairo, Egypt; 3 Core Facility Unit, College of Medicine, King Saud University, Riyadh, Saudi Arabia; 4 Organic Chemistry Department, Faculty of Science, Ain-Shams University, Cairo, Egypt; 5 Department of Botany and Microbiology, College of Science, King Saud University, Riyadh, Saudi Arabia; 6 Department of Pharmacology, College of Pharmacy, King Saud University, Riyadh, Saudi Arabia; 7 Department of Pharmaceutical Chemistry, Faculty of Pharmacy, Egyptian Russian University, Badr City, Cairo, Egypt; 8 Cancer Biology Department, Medical Biochemistry and Molecular Biology Unit, National Cancer Institute, Cairo University, Cairo, Egypt; Cairo University, Faculty of Science, EGYPT

## Abstract

*Opuntia ficus-indica* (L.) Mill. has been used in folk medicine against several diseases. The objectives of the present study were to investigate the chemical composition of the methanolic extract of *O*. *ficus-indica* (L.) Mill. flowers and their antioxidant, antimicrobial, and anticancer properties. Besides, network pharmacology and molecular docking were used to explore the potential antitumor effect of active metabolites of *O*. *ficus-indica* (L.) Mill. against breast and liver cancer. The results revealed many bioactive components known for their antimicrobial and anticancer properties. Furthermore, scavenging activity was obtained, which indicated strong antioxidant properties. The plant extract exhibited antimicrobial activities against *Aspergillus brasiliensis* (MIC of 0.625 mg/mL), *Candida albicans*, *Saccharomyces cerevisiae*, *Staphylococcus aureus*, *Escherichia coli*, and *Pseudomonas aeruginosa* at MICs of 1.25 mg/mL. The results revealed proapoptotic activities of the *O*. *ficus-indica* (L.) Mill. extract against MCF7, MDA-MB-231, and HepG2 cell lines, where it induced significant early apoptosis and cell cycle arrest at sub-G1 phases, besides increasing the expression levels of p53, cyclin D1, and caspase 3 (*p* <0.005). The network pharmacology and molecular docking analysis revealed that the anticancer components of *O*. *ficus-indica* (L.) Mill. flower extract targets the PI3K-Akt pathway. More investigations might be required to test the mechanistic pathways by which *O*. *ficus-indica* (L.) Mill. might exhibit its biological activities *in vivo*.

## Introduction

Natural products, like plant extracts, contain unlimited and diverse chemical components, which enable countless prospects for the discovery of new drugs [1]. It was reported that about 80% of the world’s population gets their primary healthcare from traditional medicine, according to data from the World Health Organization (WHO) [2]. Herbal medicines reflect a long history of human interactions with the environment, particularly in Africa. Several plant components in traditional medicine have been used to treat infectious and chronic diseases for decades [3]. Although allopathic remedies may treat different medical conditions, many individuals are returning to herbal therapies because of their availability, low prices, fewer side effects, and lower cost [3].

Because natural therapeutic agents are attractive and come in a wide variety of chemical forms from plants, animals, and microbes, the main focus of pharmaceutical research has been on finding new and effective anticancer medication from natural sources. Natural sources have been the source of more than 60% of the latest anticancer medications [4]. Based on their composition, ecology, phytochemicals, and ethnopharmacological qualities. Effective anticancer medicines have been produced in large part through the use of plants and their derivatives. Vincristine, vinblastine, etoposide, topotecan, irinotecan, podophyllotoxin, and paclitaxel are a few examples of plant derivatives. Several other plant-derived bioactive chemicals, like gimatecan and elomotecan, are in the clinical development phase as anti-cancer agents due to their unique action [5, 6]

Traditional medicine, which predominantly employs plant-based treatments, is now thought to provide primary medical care to 60% of the worldwide population and 80% of those living in developing countries. Although herbs were valued for their centuries-old medicinal, flavoring, and aromatic properties, their significance was temporarily eclipsed by synthetic items in the contemporary era. Because of the oral transmission of that knowledge from one generation to the next, it is critical to chronicle the medicinal and aromatic plants associated with traditional wisdom [7]. Additionally, the blind reliance on artificial substances has ended, and people are turning back to natural therapy in the hopes of security and safety [8].

Currently, 60% of the world’s population and 80% of those living in underdeveloped nations are believed to receive basic medical care through traditional medicine, which primarily uses plant-based remedies. In the modern age, synthetic products briefly overshadowed the importance of herbs, despite their long history of use as flavoring, aromatic, and therapeutic ingredients. The medicinal and aromatic plants, linked to traditional wisdom, should be documented since this knowledge is passed down orally from one generation to the next [7]. There has also been a shift away from the heedless dependence on synthetic drugs, with individuals returning to natural remedies for stability and safety [8].

The widespread emergence of antimicrobial resistance (AMR) due to the inappropriate use of antimicrobials has significantly limited the therapeutic options available and has had a detrimental impact on human and animal health, leading to higher rates of treatment failures and increased severity of illnesses [9]. This evolving AMR presents a global health challenge and has garnered the attention of the World Health Organization (WHO) as one of the most critical issues facing medical science. Hence, there is an urgent need to discover and develop new antimicrobial agents to combat emerging antimicrobial resistance [10–16]. Medicinal plants contain diverse chemical compounds with promising biological activities, including antimicrobial, anti-inflammatory, and antioxidant properties [17–19]. *Opuntia ficus-indica* (L.) Mill., a member of the *Cactaceae* family, has long been used in traditional medicine to treat various diseases. It is now gaining attention due to the antimicrobial activities exhibited by its bioactive compounds against numerous organisms. These compounds can be isolated from various parts of the plant, including cladodes, roots, flowers, fruits, and seeds [20–23].

Cancer remains one of the leading causes of death across genders and age groups [24]. The potential of natural products to serve as anticancer agents due to their phytochemical composition is extensively studied. These compounds demonstrate relatively high safety profiles in pharmaceutical settings and offer promising prospects for cancer treatment. Plant-derived chemicals are estimated to contribute to more than half of all anticancer drugs [25]. There is growing interest in natural compounds for chemotherapy, alongside ongoing efforts to identify novel molecular target-based compounds. *Opuntia* fruits and young stems have traditionally been used in folk medicine to treat various conditions, including burns, inflammation, nausea, allergies, diabetes, and hypertension [26, 27]. Several studies have identified well-known anticancer compounds in members of the *Cactaceae* family. Examples include *Stenocereus stellatus*, rich in the triterpene botulinic acid, which affects the proliferation of Hela cells [28], and dihydroactinidiolide and 2,4-ditert-butylphenol detected in the extract of *Pereskia bleo*, demonstrating anticancer activity against MCF-7 and A594 cells [29, 30]. Other studies have highlighted the significant anticancer properties of *O*. *ficus-indica* (L.) Mill. seed oil against adenocarcinoma cell lines Colo-320 and Colo-741 [31]. Additionally, various extracts of *O*. *ficus-indica* fruit juice have shown anticancer activities against brain cancer U87-MG, colon cancer HT-29 [32] and Caco2 [33–35], prostate cancer PC3 [35], and ovarian cancer cells (OVCA420, SKOV3) [36]. This may be attributed to the rich phytochemical composition of *O*. *ficus-indica* (L.) Mill., including malic acid, quinic acid, aconitic acid, cinnamonic acid, chlorogenic acid, p-coumaric acid, ferulic acid, and other methanolic and flavonoid compounds with well-known anticancer activities [37].

Many physiological and pathological processes in modern medicine involve complex cellular and molecular mechanisms of action that cannot be fully understood without biochemical and molecular tools [38]. In the era of artificial intelligence and large datasets, network pharmacology has emerged as a novel, multidisciplinary field at the forefront of systematic drug research. It has been widely utilized in herbal medicine research, focusing on molecular relationships between drugs and treatment objectives from a holistic system and biological network perspective [39]. Molecular docking, a tool aiding in drug design, predicts binding patterns and validates interactions between drugs and target proteins [40]. Numerous experts in herbal medicine are exploring the use of molecular docking and network pharmacology to investigate drug and disease mechanisms. This study model involves a series of assessments of the phytochemical components of herbal medicines and disease targets to anticipate key components, targets, and pathways in disease therapy using existing databases.

It’s worth noting that previous studies have primarily focused on extracts from the stems, fruits, seeds, and cladodes of *O*. *ficus-indica* (L.) Mill., with fewer investigations concerning its flower parts. Moreover, some studies overlooked certain cancers, such as liver cancer, while focusing mainly on colon and breast cancer. The current study provides a novel investigation of the methanolic extract of *O*. *ficus-indica* (L.) Mill. flowers, with an emphasis on their antibacterial, antioxidant, and anticancer effects. While prior studies focused on the stems, fruits, seeds, and cladodes, this study delves into the lesser-known flower components. Furthermore, the integration of network pharmacology and molecular docking analysis focuses on breast and liver cancer pathways, particularly the PI3K-Akt pathway. This combination method sheds fresh light on the bioactive components of *O*. *ficus-indica* (L.) Mill. flowers and their potential therapeutic applications, filling gaps in the existing literature. Additionally, the current study aims to explore the antimicrobial potential of methanol extracts of *O*. *ficus-indica* (L.) Mill. against some selected bacterial and fungal species.

## Materials and methods

### Collection of *O*. *ficus-indica* (L.) Mill. flowers

Flowers of *O*. *ficus-indica* (L.) Mill. were harvested in May 2022 (the blossoming date) from the Giza governorate, Egypt. Latitude and longitude coordinates are 30° 0’ 47.0016’’ N, and 31° 12’ 31.8708’’ E, respectively. For biological and phytochemical investigations, the samples were individually air-dried in sheds, ground into a powder, and stored in tight-sealed round flasks. The Botany Department, Faculty of Science, Cairo University, Egypt, verified the plant’s identity.

### *O*. *ficus-indica* (L.) Mill. flower parts for extraction

1.5 kg of the air-dried flowers were ground into a coarse powder, dissolved in petroleum ether, and then extracted one at a time using a maceration method with 70% methanol (5×2L) at room temperature until the material was completely extracted. After gathering the filtrates, they were vacuum-dried at 40°C to yield 60 g of net weight (w/w) [41].

### Gas chromatography-mass spectrometry (GC-MS)

The organic extract of *O*. *ficus-indica* (L.) Mill. was analyzed using the 5977C GC/MSD instrument equipped with a polar (DB-Wax) column and MSD-5975C detector (Agilent Technologies, Santa Clara, CA, United States). The machine is equipped with a split-spitless injector at the split ratio of 10:1, a Quick-Swap assembly, an HP-5MS fused silica capillary column (5% phenyl/95% dimethylpolysiloxane, 30 m × 0.25 mm i.d. with 0.25 *μ*m film thickness), and an Agilent autosampler model 7693 (Agilent Technologies, Santa Clara, CA, United States). Sample preparation and processing were according to the manufacturer’s instructions. The relative composition of the extract components was determined based on the peak area measured by the HP-5MS column without the correction factor. The separated compounds were identified by searching the library of the National Institute of Standards and Technology (NIST14) at https://chemdata.nist.gov/ and the NIST Mass Spectral Search Program (Version 2.2) as described before [42]. The chemical structures of the resulting compounds were drawn by the web-based data visualization platform MolView v2.4 (https://molview.org/), while the molecular weights and chemical formulas were obtained from the chemicals database of PubChem (https://pubchem.ncbi.nlm.nih.gov/). Also, the chemical classification of compounds was obtained by the web-based application ClassyFire: https://cfb.fiehnlab.ucdavis.edu/ [43].

### Antioxidant activity by free radical scavenging (DPPH) assay

The organic extract of *O*. *ficus-indica* (L.) Mill. was tested for its possible antioxidant activity using the chemical compound 2, 2-diphenyl-1-picrylhydrazyl (DPPH) [44]. Briefly, serial dilutions (0, 0.75, 1.5, 3, 6, 12, 25, 50, and 100 μg/mL) were prepared with distilled water for each extract or ascorbic acid that acts as a positive control in a 96-well plate. Then, 150μl of the DPPH solution (0.1 mM) was added to each well and incubated in the dark for 30 min. Later, the colorimetric changes were assessed at 517 nm by the microplate reader Synergy 2 (BioTek Inc., Vermont, United States). The inhibition percentage of DPPH free radical scavenging activity was calculated at the average of triplicates as follows:

$$\%\;\text{scavenging}\;\text{activity}=\left(\text{Ac}-\text{At}\right)\text{/Ac}\times 100$$

Where “Ac” is the absorbance of DPPH (concentration 0 μg/mL) and “At” is the absorbance of the sample (extract or ascorbic acid). The experiment was repeated in triplicate, and average percentages were calculated.

### Antimicrobial activity of *O*. *ficus-indica* (L.) Mill. flower parts

#### Microbial strains and culture conditions

The antimicrobial activity of *O*. *ficus-indica* (L.) Mill. flower extract was tested against six strains of bacteria and fungi: *S*. *aureus* (ATCC 25923), *E*. *coli* (ATCC 25922), *P*. *aeruginosa* (ATCC 27853), *C*. *albicans* (ATCC 10231), *S*. *cerevisiae* (ATCC 9763), and *A*. *brasiliensis* (ATCC 16404). No clinical isolates were tested in this study.

The tested organisms were prepared before antimicrobial testing as described by Ramírez-Moreno et al. (2017) with some modifications [45]. Except for *A*. *brasiliensis*, which was incubated at 25°C ± 1 for 48 hours, the microorganisms were inoculated into 100 mL of tryptic soy broth medium and incubated at 35°C ± 1. Afterwards, a fresh culture was created by streaking a loopful of broth on Sabaroud Dextrose Agar for fungi and Tryptic Soy Agar for bacteria. Additionally, bacterial suspensions were prepared by inoculating 3–4 colonies into sterile saline with an adjustment of turbidity equivalent to 0.5 McFarland using a DensiCHEK© optical device. That adjustment results in a suspension containing approximately 1–2 x 10^8^ CFU/mL. These suspensions were diluted by 5% in Muller-Hinton broth (MHB), which resulted in an approximate concentration of 1.0 × 10^6^ CFU/mL.

#### Determination of the minimal inhibition concentration (MIC)

The MICs of the extracts were determined using the microdilution method. First, a stock solution of the plant extract was prepared by adding 100 μl of condensed extract to 900 μl of dimethyl sulfoxide (DMSO) [46]. This was serially diluted to obtain various concentrations ranging from 0.019 to 10 mg/ml. A 10 μl aliquot of bacterial suspensions was put on a sterile 96-well plate containing 100 μl of MHB and 100 μl of serial dilutions of the extract. A positive control (without extract) and a negative control (broth only) were included on each microplate. The incubation was done at 35°C for 24 hours, except for *A*. *brasiliensis*, which was incubated at 25°C for 48 hours. After incubation, wells were checked for any visual growth, and the optical density of the inoculated wells was measured at 600 nm and 340 nm for bacteria and fungi, respectively. The MIC was read in triplicate.

#### Determination of the minimal bactericidal and fungicidal concentration (MBC and MFC)

MBC was determined by streaking 10 μl of broth from those wells with no visible growth or turbidity on the Muller-Hinton agar plate. After incubation, the plates are examined for growth. The MBC and MFC were defined as the minimum concentration of extract that prevents the growth of microbes on culture media [47].

### Anticancer activities of *O*. *ficus-indica* (L.) Mill. flower parts

#### Human cell culture

In the current study, three cell lines were used for the determination of the anticancer activity of the methanolic extract of *O*. *ficus-indica* (L.) Mill. The cells included the human breast cancer cell lines MCF-7, MDA-MB-123, and the hepatocellular carcinoma cell line (HepG2). The cells were purchased from the American Type Culture Collection (ATCC) (Manassas, VA, United States). All cells were maintained in Dulbecco’s Modified Eagles Medium (DMEM) (GIBCO, Thermo Fischer Scientific, NY, United States), which was supplemented with 10% fetal calf serum (FCS), 1% penicillin/streptomycin, and 1% L-glutamine (GIBCO, Thermo Fischer Scientific, NY, United States). The cells were incubated at 37°C in a CO_2_ incubator. The culture medium was changed every three days (subculture), and all culture work was performed in a biological safety cabinet under aseptic techniques.

#### Cell viability assay by SRB (Sulforhodamine-B)

The sulforhodamine-B (SRB) method was applied to determine cytotoxicity [48]. Cells were seeded at a concentration of 4 × 10^3^ cells/well in 96-well microtiter plates, and after 24 hours, they were allowed to attach before being incubated with *O*. *ficus-indica* (L.) Mill. The cells were then treated with various concentrations (0, 10, 50, 75, 100, 400, 800, and 1000 μg/mL) of *O*. *ficus-indica* (L.) Mill. extract for 48 hours using paclitaxel as positive control. The optical density (O.D.) of each well was measured spectrophotometrically at 570 nm using an ELISA microplate reader (TECAN Sunrise^™^, Germany). The mean values were estimated as the percentage of cell viability as follows: O.D. (treated cells) / O.D. (control cells) × 100. The IC_50_ value (the concentration that produces 50% inhibition of cell growth) of plant extract was calculated using dose-response curve-fitting models (Graph-Pad Prism software, version 7).

#### Apoptosis assay

The apoptotic effect of the methanolic extract of *O*. *ficus-indica* (L.) Mill. was assessed by the FITC Annexin V Apoptosis Detection Kit with PI (BioLegend Co., San Diego, CA, United States). After calculating the IC_50_, cells were treated with the methanolic extract at the IC_50_ concentration and compared to the untreated cells. The following day, 0.25% trypsin/EDTA (GIBCO, Thermo Fischer Scientific, NY, USA) was used to collect the cells after they had been washed with PBS. The cells were then incubated for 15 minutes on ice in the dark using a combination of PI and Annexin V-FITC. The apoptosis/necrosis was detected by the BD FACSCanto II cell analyzer (BD Biosciences, CA, United States) at an emission of 530 nm (for Annexin V FITC) and >575 nm (for PI). A dot plot was used to estimate the percentage of different cellular statuses as follows: the lower right corner expressed early apoptosis (positive in annexin V-FITC), the upper left expressed necrosis (positive for PI), the upper right showed the dead cells/late apoptosis (positive in both annexin V-FITC and PI), and the lower right showed the live cells (negative in both annexin V-FITC and PI) [49]. The experiment was repeated in triplicate, and average percentages were calculated.

#### Cell cycle assay

Cell cycle analysis represents an early methodology utilizing PI reagents for the univariate examination of cellular DNA via flow cytometry. Cells were exposed to the methanolic extract at the IC_50_ concentration and juxtaposed with untreated cells. In brief, following a 24-hour incubation period, cells were rinsed with phosphate-buffered saline (PBS) and detached using trypsin/EDTA (GIBCO, Thermo Fischer Scientific, NY, United States). Subsequently, the cells were fixed with 1 mL of 70% ice-cold ethanol for 30 minutes on ice. Afterwards, cells were washed with ice-cold PBS, incubated with RNase A (10 mg/mL) (Sigma-Aldrich, St. Louis, MO, USA), and PI (50 μg/mL) at room temperature for 10 minutes. The resulting stained cells’ cell cycle arrest was assessed using the BD FACSCanto II cell analyzer (BD Biosciences, CA, United States) with emission >575 nm. The outcomes were depicted via a histogram, delineating distinct regions corresponding to the G0/G1, S, and G2/M phases, and the sub-G1 phase indicative of dead cells. The cell counts, at all phases, were collectively compared [50]. The experiment was replicated in triplicate, and average percentages were computed.

#### Reactive Oxygen Species (ROS) analysis

As previously mentioned, the efficacy of the methanolic extract of *O*. *ficus-indica* (L.) Mill. as a prooxidant was evaluated by screening the ROS generation using the DCFDA/H_2_DCFDA Cellular ROS Assay Kit (ABCAM, Cambridge, UK) and flow cytometer [51]. Briefly, the treated and non-treated cells were incubated for 24 hours, then harvested and washed with PBS, as previously mentioned. Later, the cells were treated with 2’,7’-dichlorofluorescin diacetate (DCFDA) and kept at 37°C for 30 minutes. After washing twice with PBS, the cells were analyzed by flow cytometer at an emission of 530 nm (FL1 channel) and 150 mV [50]. The experiment was repeated in triplicate.

#### Western blot analysis

The impact of the methanolic extract of *O*. *ficus-indica* (L.) Mill. on various cellular proteins was investigated. Cells were plated at a density of 1 × 10^6^ cells per plate in a DMEM-complete medium and incubated in a 5% CO_2_ atmosphere at 37°C until reaching 50% confluence. Subsequently, cells were treated with the methanolic extract at the IC_50_ concentration, in comparison to untreated cells, and incubated for 24 hours. On the day of detection, cells were washed with ice-cold PBS, harvested, and incubated on ice with radioimmunoprecipitation assay buffer (RIPA buffer) (Santa Cruz Biotechnology, Inc., Dallas, TX, United States) for 30 minutes on an orbital shaker. The resulting protein lysate was separated and quantified using the colorimetric Bradford protein assay (ABCAM, Waltham, MA, United States) at an absorbance of 595 nm. Subsequently, equal volumes of protein lysates were subjected to sodium dodecyl sulfate-polyacrylamide gel electrophoresis (10% Precast SDS-PAGE gel) at 100 V for one hour. The separated proteins were then transferred onto a polyvinylidene fluoride (PVDF) membrane using a semi-dry method. The PVDF membrane was blocked with 5% non-fat dried milk for one hour and subsequently incubated with various primary antibodies targeting cellular proteins such as p53, cyclin D1, caspase 3, and β-actin. Following incubation, the membranes were probed with Luminol HRP chemiluminescence substrate (Santa Cruz Biotechnology, Inc., Dallas, TX, USA) and visualized using a c-digit blot scanner (LI-COR, Nebraska, United States). The resulting band intensity was quantified using ImageJ software version 1.51.8 (National Institutes of Health, USA). The experiment was conducted in triplicate.

### Network pharmacology

#### Screening of active ingredients from *O*. *ficus-indica* (L.) Mill. and gathering their targets

The retrieval of 2D structure files (SDF), PubChem IDs, and SMILES (S1 Table) for five active ingredients, representing the primary and predominant metabolites *of O*. *ficus-indica* (L.) Mill., previously identified via LC-MS and MS/MS, was performed utilizing the PubChem database (https://pubchem.ncbi.nlm.nih.gov/) [52]. These active metabolites (2D structure files) underwent further filtration based on their drug-likeness (QED > 0.3) and oral bioavailability (Lipinski’s rule) using ADMET Lab 2.0 (https://admetmesh.scbdd.com/service/evaluation/index [53] (S1 Table). Additionally, their potential biological targets were predicted through the BindingDB server (https://www.bindingdb.org/rwd/bind/index.jsp) [54] and the Swiss target prediction web tool (http://www.swisstargetprediction.ch/?) [55] (S2 Table). Subsequently, the compiled biological targets (261 targets) were annotated using the UniProt database (https://www.uniprot.org/) [56] to obtain the UniProt IDs corresponding to their genes (S2 Table).

#### Gathering of breast cancer target genes and prediction of active ingredients for breast cancer treatment

The DisGeNET database (https://www.disgenet.org/) was queried for genes associated with cancer, specifically using the search terms breast cancer and liver cancer (S3 and S4 Tables) [57]. Employing the Venn diagram intersection feature of FunRich 3.1.3 software, the data retrieved for genes linked to breast cancer (2579 genes annotated with UniProt) and liver cancer (1543 genes annotated with UniProt) were juxtaposed with the previously predicted target diseases of active metabolites from *O*. *ficus-indica* (L.) Mill. (261 targets) [58].

#### Constructing a protein-protein interaction network

The STRING database (https://cn.string-db.org/) was utilized to acquire the Protein-Protein Interaction (PPI) network, and further analysis and enhancement were performed using Cytoscape 3.9.0 software. Initially, potential therapeutic targets for breast and liver cancers were input into the STRING online database, with parameters and constraints set (species limited to Homo sapiens, and filtered through a combined score ≥ 0.95 as the threshold). The PPI network results were downloaded in TSV format and imported into Cytoscape 3.9.0 software to create a network diagram for visualization. Using the CytoHubba plug-in, each node in the network was calculated, and core target genes were identified through screening.

#### Gene Ontology and Kyoto Encyclopedia of Genes and Genomes pathway enrichment analysis

The Gene Ontology (GO) enrichment analysis was performed using FunRich 3.1.3 software to identify significant GO terms. GO terms with *P-values* below 0.05 and enrichment scores exceeding 5 were considered noteworthy and subjected to further analysis. Kyoto Encyclopedia of Genes and Genomes (KEGG) pathway analysis results were evaluated using ShinyGO 0.77 (http://bioinformatics.sdstate.edu/go/) [59], employing an adjusted *P-value* threshold of 0.05 for pathway identification. Visualization analysis, including creating bubble charts and histograms, was conducted using the online tools available on the bioinformatics platform (http://www.bioinformatics.com.cn/).

### Molecular docking

Molecular docking studies were conducted to investigate the binding affinity of *O*. *ficus-indica* (L.) Mill. metabolites against the target enzyme, PI3K. The MOE software version 2019.0102 was utilized for molecular docking simulations [60]. The database of active metabolites underwent energy minimization, hydrogen addition, and partial charge calculation for preparation, followed by saving in mdb extension format [61]. The target enzyme, PI3K, was retrieved from the Protein Data Bank (www.rcsb.org) with PDB ID: 5xgj and underwent preparation and validation through MOE’s automatic quick preparation tool. Docking simulations were performed using Amber10 Forcefield, and ligand-protein complex interactions were evaluated through pose visualization and scoring functions [62]. Docking studies were validated by assessing the root mean square deviation (RMSD) values for co-crystalized ligand-protein isozymes (PI3K: 1.2683).

## Results

### The phytochemical analysis and antioxidant activity of the methanolic extract of *O*. *ficus-indica* (L.) Mill. flowers

**Fig 1:**
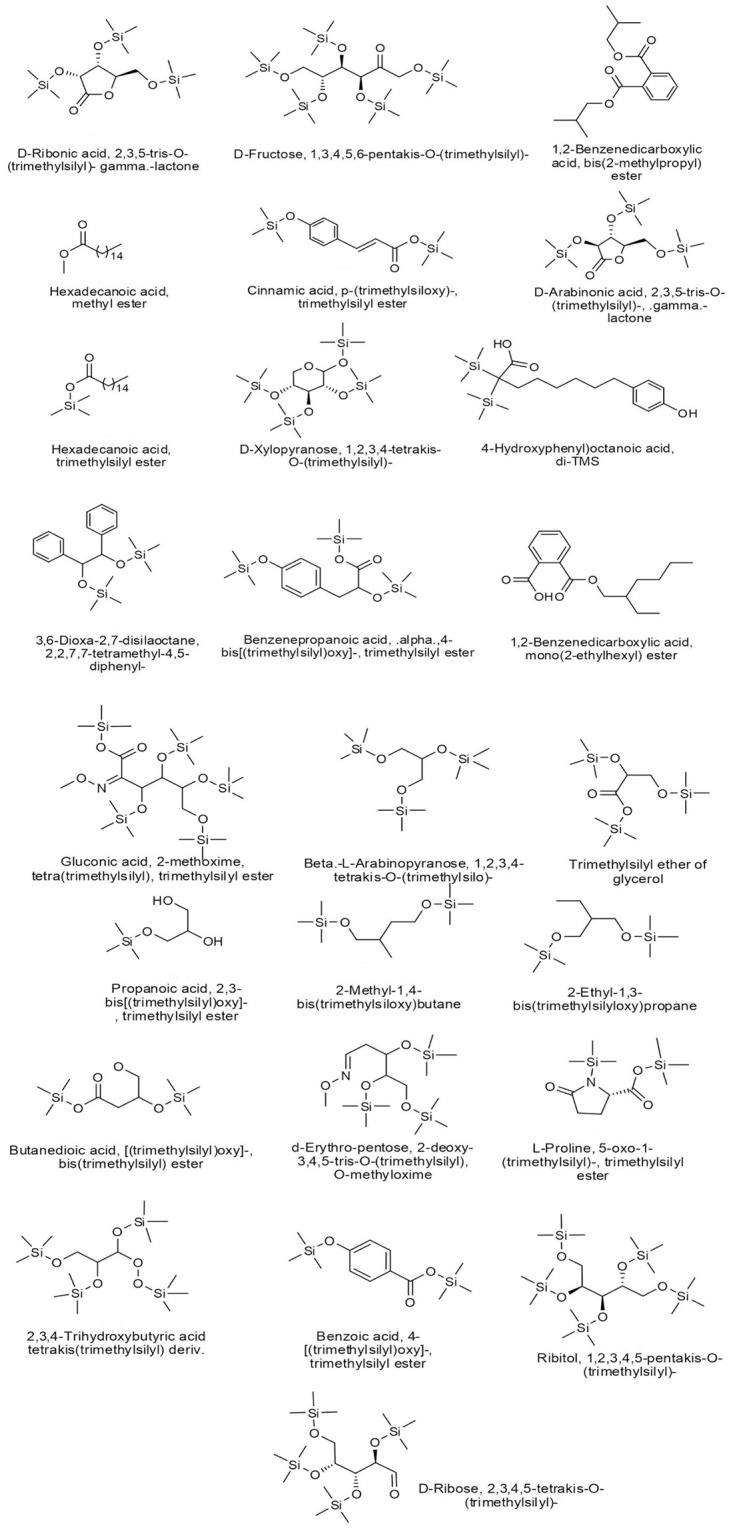
Structures of the methanolic compounds obtained from the GC-MS analysis.

In the current study, the GC-MS analysis revealed that the methanolic extract of *O*. *ficus-indica* (L.) Mill. flowers contain many bioactive components, which are known for their antimicrobial and anticancer properties. As shown in Table 1, there was a wide distribution of organic compounds, which included derivatives of gluconic acid, glyceric acid, malic acid, threonic acid, D-ribose, and hexadecanoic acid. Also, a wide variety of monosaccharides, carbohydrates, and carbohydrate conjugates were estimated at a retention time (RT) of 7.401, 8.083, 10.642, 11.655, and 13.212 minutes. Other metabolites included some aromatic methanolic compounds such as benzoic acid derivatives (Vanillic acid, Isobutyl phthalate, Monoamyl phthalate, and Phenyllactic acid derivatives), fatty acids (hexadecanoic acid, octanoic acid, and Phenyllactic acid derivatives), lactones, and other methanolic derivatives such as p-Coumaric acid (Fig 1).

**Table 1:** Chemical composition of the methanolic extract of *O*. *ficus-indica* (L.) Mill. flowers.

RT (min)	Area (Ab*s)	Formula	Name	Classification	MW (Amu)	%
**6.332**	2888539	C_22_H_53_NO_7_Si_5_	Gluconic acid, 2-methoxime, tetra(trimethylsilyl)-, trimethylsilyl ester	Organosilicon compound	583.267	2.4
**7.401**	568174	C_17_H_42_O_5_Si_4_	Beta-L-Arabinopyranose, 1,2,3,4-tetrakis-O-(trimethylsilo)	Monosaccharides	438.211	0.5
**7.526**	50665619	C_12_H_32_O_3_Si_3_	1,2,3-tris-(trimethylsilanyloxy)-propane	Organosilicon compounds	308.166	42.5
**8.083**	411829	C_12_H_30_O_4_Si_3_	Propanoic acid, 2,3-bis[(trimethylsilyl)oxy]-, trimethylsilyl ester	Carbohydrates and carbohydrate conjugates	322.145	0.4
**8.44**	1036103	C_11_H_28_O_2_Si_2_	2-Methyl-1,4-bis(trimethylsiloxy)butane	Organosilicon compounds	248.163	1
**8.74**	4000589	C_11_H_28_O_2_Si_2_	2-Ethyl-1,3-bis(trimethylsilyloxy)propane	Organosilicon compounds	248.163	3.4
**9.447**	1799823	C_13_H_30_O_5_Si_3_	Butanedioic acid, [(trimethylsilyl)oxy]-, bis(trimethylsilyl) ester	Organosilicon compounds	350.14	1.5
**9.622**	1094692	C_15_H_37_NO_4_Si_3_	d-Erythro-pentose, 2-deoxy-3,4,5-tris-O-(trimethylsilyl)-, O-methyloxime	Organosilicon compounds	379.203	0.9
**9.76**	3745410	C_11_H_23_NO_3_Si_2_	L-Proline, 5-oxo-1-(trimethylsilyl)-, trimethylsilyl ester	Amino acid derivative	273.122	3.1
**10.028**	382522	C_16_H_40_O_5_Si_4_	2,3,4-trihydroxybutyric acid tetrakis (trimethylsilyl) derivative.	Organosilicon compounds	424.195	0.4
**10.479**	1461908	C_14_H_24_O_4_Si_2_	Benzoic acid, 4-[(trimethylsilyl)oxy]-, trimethylsilyl ester	Benzoic acids and derivatives	282.111	1.2
**10.642**	271543	C_20_H_52_O_5_Si_5_	Ribitol, 1,2,3,4,5-pentakis-O-(trimethylsilyl)	Monosaccharides	512.266	0.3
**10.804**	1584738	C_18_H_45_NO_5_Si_4_	D-Ribose, 2,3,4,5-tetrakis-O-(trimethylsilyl)	Organosilicon compounds	438.211	1.3
**11.292**	4815624	C_14_H_32_O_5_Si_3_	D-Ribonic acid, 2,3,5-tris-O-(trimethylsilyl)-, gamma.-lactone	Gamma-Butyrolactones	364.156	4
**11.655**	15775347	C_22_H_55_NO_6_Si_5_	D-Fructose, 1,3,4,5,6-pentakis-O-(trimethylsilyl)-	Monosaccharides	540.261	13.2
**11.999**	1938379	C_16_H_22_O_4_	1,2-Benzenedicarboxylic acid, bis(2-methyl propyl) ester	Benzoic acids and derivatives	278.152	1.6
**12.23**	2357663	C_17_H_34_O_2_.	Hexadecanoic acid, methyl ester	Fatty acid esters	270.256	2
**12.374**	883009	C_15_H_24_O_3_Si_2_	Cinnamic acid, p-(trimethyl siloxy)-, trimethylsilyl ester	Cinnamic acids and derivatives	308.126	0.7
**12.581**	1084906	C_14_H_32_O_5_Si_3_	D-Arabinonic acid, 2,3,5-tris-O-(trimethylsilyl)-, gamma. -lactone	Lactones	364.156	0.9
**12.825**	2659800	C_19_H_40_O_2_Si	Hexadecanoic acid, trimethylsilyl ester	Organosilicon compounds	328.28	2.2
**13.212**	2319358	C_17_H_42_O_5_Si_4_	D-Xylopyranose, 1,2,3,4-tetrakis-O-(trimethylsilyl)-	Monosaccharides	438.211	1.9
**13.7**	6429101	C_20_H_36_O_3_Si_2_	(4-hydroxyphenyl) octanoic acid, di-TMS	Fatty acid, Phenoxy compounds	380.22	5.4
**13.825**	2246851	C_20_H_30_O_2_Si_2_	3,6-Dioxa-2,7-disilaoctane, 2,2,7,7-tetramethyl-4,5-diphenyl-,-	Organosilicon compounds	358.178	1.9
**14.489**	2157183	C_19_H_36_O_5_Si_3_	Benzenepropanoic acid, alpha.,4-bis[(trimethylsilyl)oxy]–, trimethylsilyl ester	Fatty acid, Phenoxy compounds	398.176	1.8
**15.102**	6580849	C_13_H_16_O_4_	1,2-Benzenedicarboxylic acid, mono(2-ethylhexyl) ester	Benzoic acids and derivatives	278.152	5.5

### Antioxidant activity

**Fig 2:**
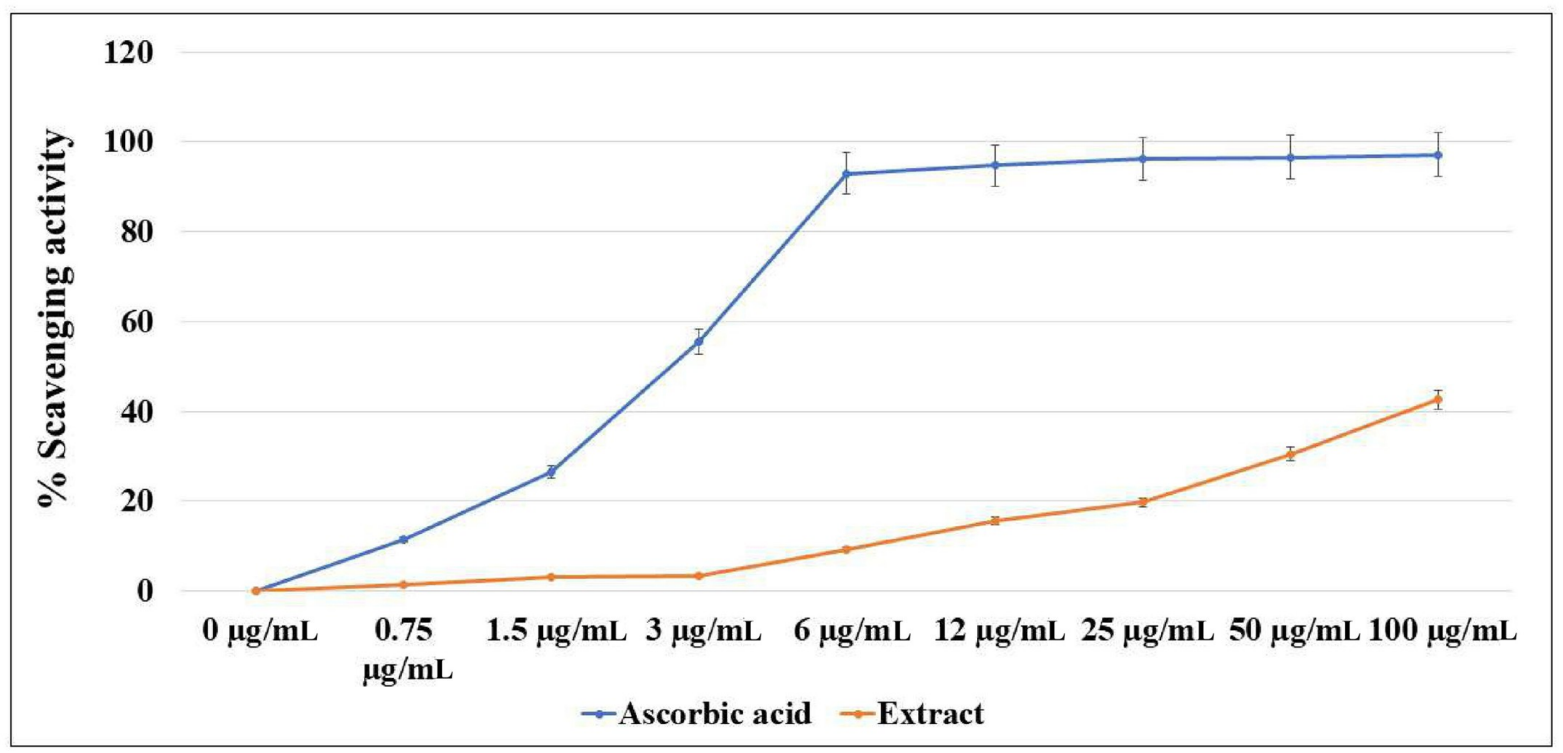
DPPH assay of the methanolic extract of *O*. *ficus-indica* (L.) Mill. flowers.

The antioxidant properties of *O*. *ficus-indica* (L.) Mill. were estimated versus the ascorbic acid using a DPPH assay (Fig 2). The obtained results showed strong antioxidant activity for the methanolic extract. The scavenging activity was noticed at concentrations > 6 μg/mL, where the highest scavenging activity (41%) was recorded at the concentration of 100 μg/mL, compared to 98% at the same concentration of ascorbic acid.

### Antimicrobial activity

The methanolic extract of *O*. *ficus indica* (L.) Mill. flowers exhibited remarkable antimicrobial activity against the tested bacteria and fungi. The extract was most effective against *Aspergillus brasiliensis*, which inhibited growth at MIC 0.625 mg/mL and was killed at MFC 2.5 mg/mL. The optimum MICs against other tested fungi (*Candida albicans* and *Saccharomyces cerevisiae*) and bacteria (*Staphylococcus aureus*, *Escherichia coli*, and *Pseudomonas aeruginosa*) were obtained at a concentration of 1.25 mg/mL. The MBCs and MFCs obtained at a concentration of 5 mg/mL was optimal for *S*. *aureus*, *E*. *coli*, *P*. *aeruginosa*, and *S*. *cerevisiae*, while for *C*. *albicans*, it was 2.5 mg/mL. Indeed, it is evident that the extract has better antifungal than antibacterial activity (Table 2).

**Table 2:** MBC and MIC of different strains treated with the methanolic extract of *O*. *ficus indica* (L.) Mill. flowers.

Organism	MICs (mg/mL)	MBCs and MFCs (mg/mL)
***E*. *coli***	1.25	5
***P*. *aeruginosa***	1.25	5
***S*. *aureus***	1.25	5
***S*. *cerevisiae***	1.25	5
***C*. *albicans***	1.25	2.5
***A*. *brasiliensis***	0.625	2.5

### Anticancer properties

#### Cytotoxic activity

**Fig 3:**
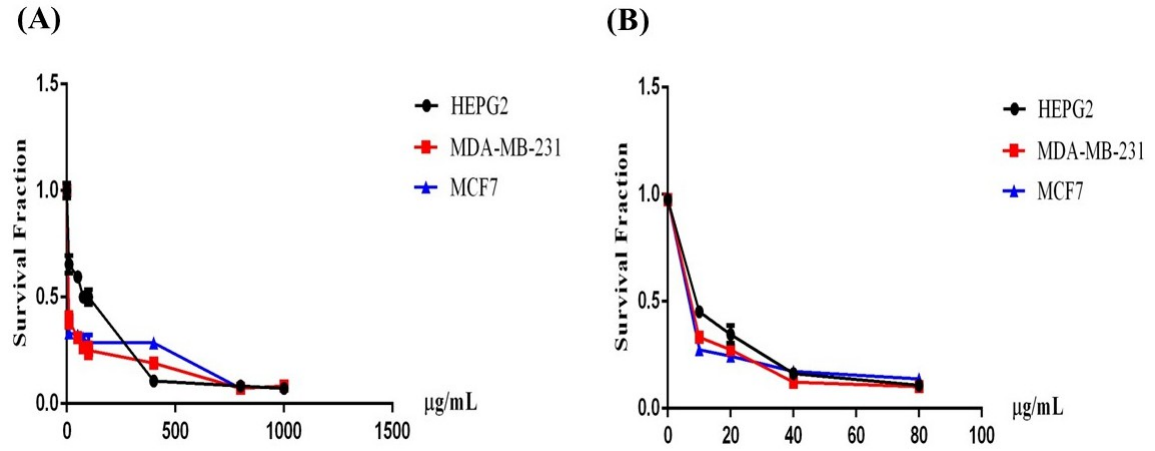
Cytotoxic activity of (A) *O*. *ficus-indica* (L.) Mill. and (B) Paclitaxel as a positive control. The IC_50_ was evaluated against MCF7, MDA-MB-231, and HEPG2 cell lines.

In the current study, the anticancer properties of the methanolic extract of *O*. *ficus-indica* (L.) Mill. flowers were evaluated against three cell lines: MCF-7 (breast), MDA-MB-231 (breast), and HepG2 (hepatocellular carcinoma) cells. The cytotoxic analysis of the cells treated with serial dilutions of the extract revealed a significant decline in cellular viability, while the cellular toxicity levels were highest at the highest concentration (Fig 3). The inhibition concentrations at 50% of cellular viability (IC_50_) were calculated for all cell lines. The results showed that MCF-7 and MDA-MB-231 had IC_50_s of 10 μg/mL and 5 μg/mL, respectively, whereas HepG2 cells had a higher IC_50_ of 80 μg/mL (Fig 3A). Paclitaxel, which was used as a positive control, showed IC_50_ values of 6.3 μg/mL, 7 μg/mL, and 9.3 μg/mL against MCF7, MDA-MB-231, and HEPG2, respectively (Fig 3B).

#### Apoptosis assay

**Fig 4:**
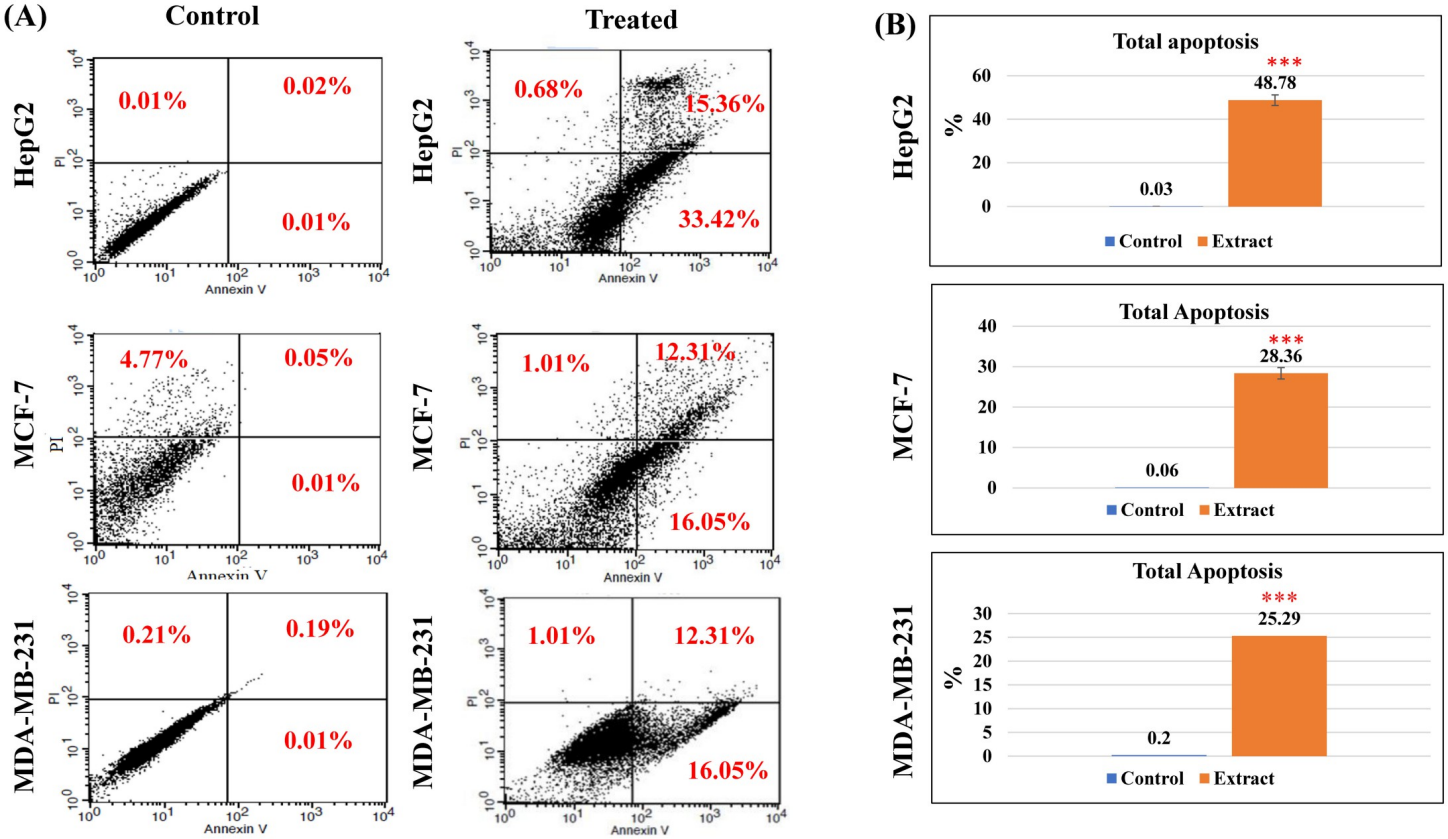
Annexin V/PI assay of the methanolic extract *of O*. *ficus-indica* (L.) Mill. flowers against different cancer cell lines. (A) Dot plots of the flow cytometric analysis of the treated and untreated cells; the quadrant was set to indicate the live cells (lower left corner), early apoptosis (lower right corner), late apoptosis (upper right corner), and necrosis (upper left corner). (B) A graphic bar chart to show the percentages of total apoptosis (early and late apoptosis) of IC_50_-treated and untreated cells. *** indicated the statistical significance of the results at a *P-value* <0.001 compared to control untreated cells.

The proapoptotic properties of the plant extract were assessed by flow cytometry to investigate the apoptotic or necrotic behavior using the principle of Annexin V for the detection of apoptosis level and propidium iodide (PI) for necrosis (Fig 4). For each cell line, the cells treated with the extract at the IC_50_ concentration were compared to the untreated cells. As shown in Fig 4A, the results revealed that the extract induced significant early apoptosis in all cell lines. Furthermore, for HepG2 and MCF-7 cells, a portion of the cells underwent late apoptosis behavior or was completely dead at the IC_50_ dose. The total apoptotic effects were calculated for each cell line by combing both early and late apoptosis percentages as follows; HepG2 (~49%), MCF-7 (28%), and MDA-MB-231 (25%) (Fig 4B).

#### Cell cycle assay

**Fig 5:**
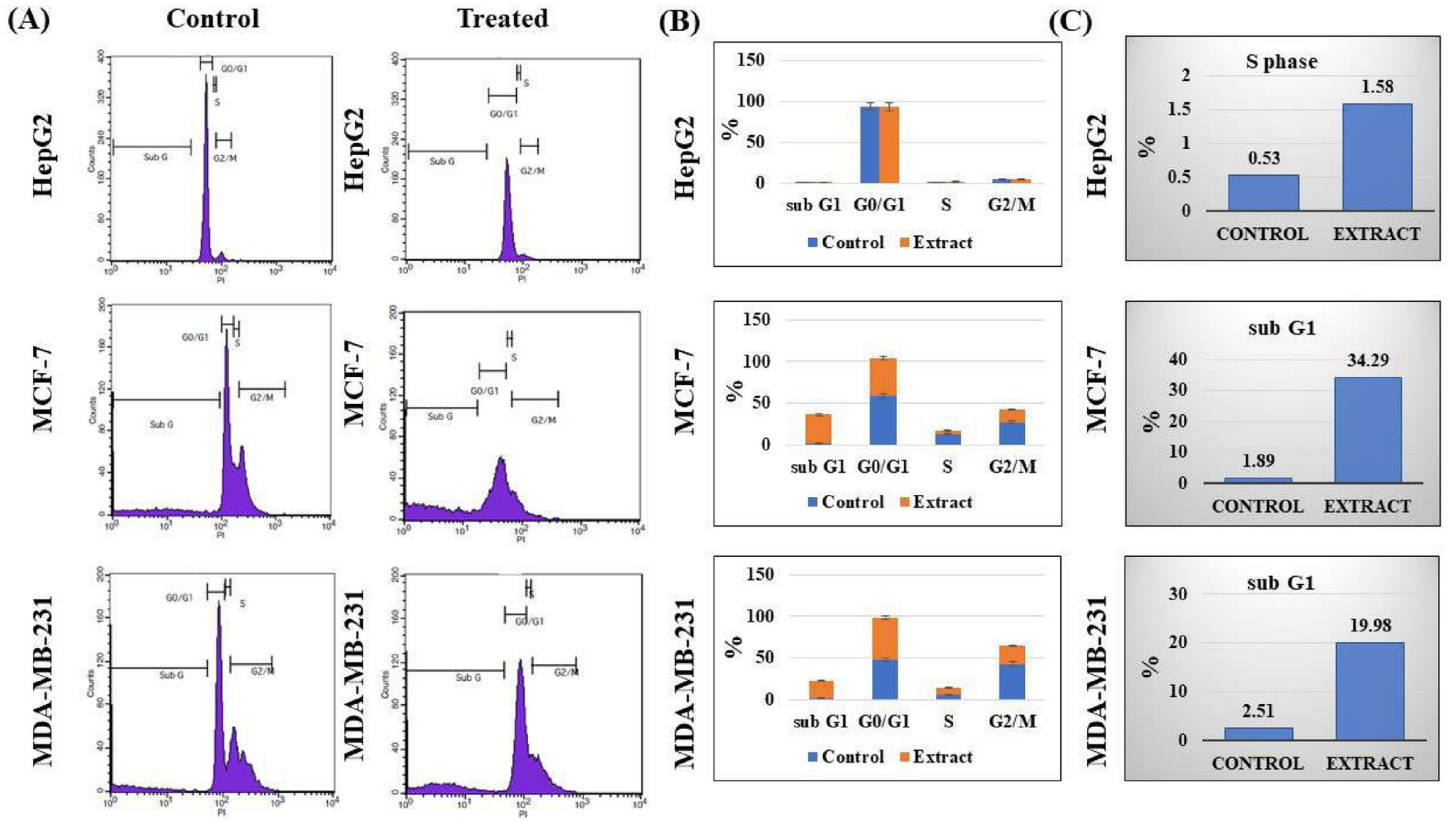
Cell cycle assay of the methanolic extract *of O*. *ficus-indica* (L.) Mill. flowers against different cancer cell lines. (A) Histograms of the flowcytometric analysis of the treated and untreated cells; four areas were marked to indicate the cell cycle stages of G0/G1, S, and G2/M phases, in addition to the sub-G1 region. (B) A graphic bar chart to express the percentages of all cell cycle stages of the treated and control cells. (C) A graphic bar chart to express the percentages of cell cycle stages included in the cellular arrest for each cell line.

To study if *O*. *ficus-indica* (L.) Mill. was able to induce any significant cell cycle arrest in the tested cells, the cell cycle analysis was evaluated using the PI staining protocol and flow cytometry (Fig 5). A significant increase in the levels of the S phase was noticed in the treated HepG2 cells at 1.58% compared to 0.53% in the control cells (*p* < 0.05). In the breast cancer cell lines, we couldn’t detect any significant cell cycle arrest; however, there were strong elevations of the sub-G1 levels. That might illustrate the cytotoxic activity of that extract in the tested cell lines.

#### ROS assay

**Fig 6:**
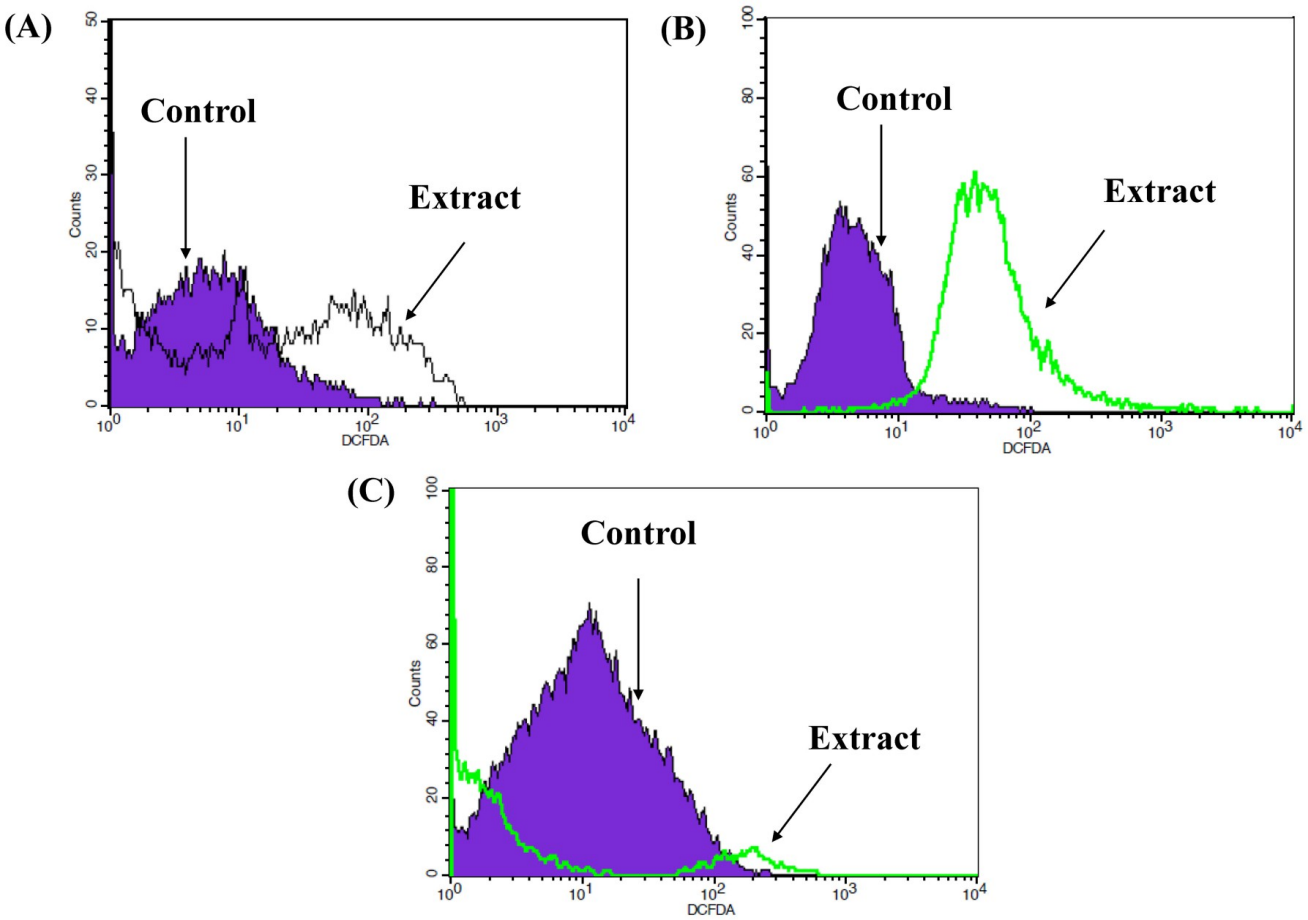
ROS assay of the methanolic extract of *O*. *ficus-indica* (L.) Mill. flowers against different cancer cell lines. DCFDA and flow cytometry were used to assess the oxidative stress in the cells treated with the IC_50_ concentration of the plant extract. (A) HepG2; (B) MCF-7; (C) MDA-MB-231.

ROS analysis was used to evaluate the ability of the methanolic extract of *O*. *ficus-indica* (L.) Mill. flowers to act as a prooxidant agent against cancer cells by increasing ROS production. The results showed a clear shift in the DCFDA histogram peaks of the treated HepG2 and MCF-7 cells compared to the untreated control. That indicated the oxidative stress by increasing the early production of ROS at 24 hours, which reduced the non-fluorescent H_2_DCFDA compound to a green fluorescent DCFDA compound (Fig 6). MDA-MB-231 had a very slight shifting of the fluorescent DCFDAA compared to the untreated cells.

#### Western blot analysis

**Fig 7:**
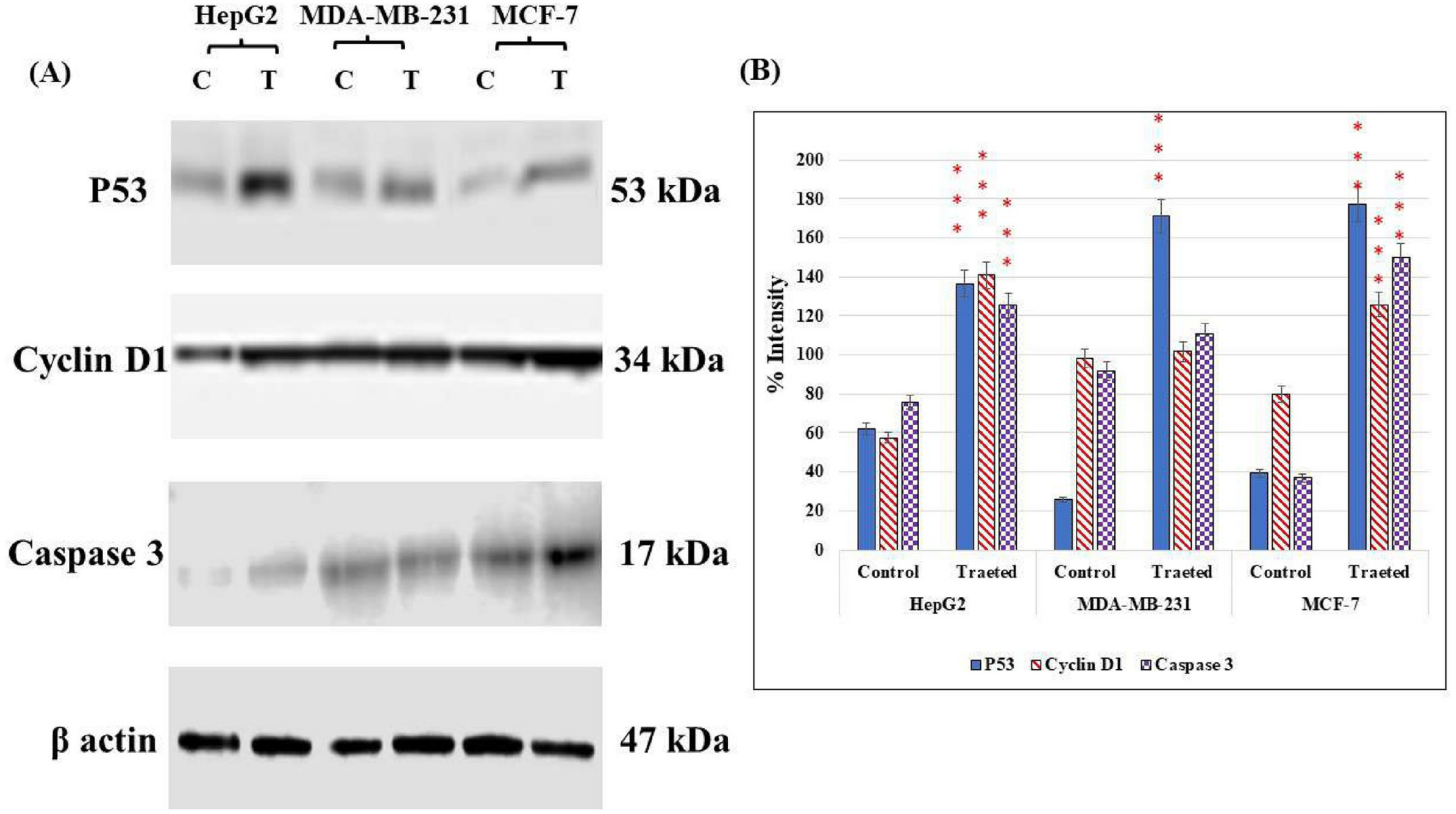
Immunoblotting analysis of cells treated with the methanolic extract of *O*. *ficus-indica* (L.) Mill. flowers at the IC_50_ concentration. (A) Western blot results for each cell line. (B) A graphic bar chart to show the changes in the intensity levels of the studied proteins in the IC_50_-treated and untreated cells. *** indicated the statistical significance of the results at a *P* <0.001 compared to control untreated cells.

To validate the above-mentioned results of the anticancer activities of *O*. *ficus-indica* (L.) Mill. flowers, western blot analysis was used to investigate the change in the expression levels of some cellular proteins involved in the apoptotic pathway. The immunoblotting analysis was performed against the tumor suppressor protein p53, the apoptotic mediator protein caspase 3, and the regulator of cell cycle progression, cyclin D1. All experiments were evaluated against the housekeeping protein β-actin (Fig 7). In HepG2 and MCF-7, the plant extract drove the cells towards apoptosis by increasing the expression levels of p53. The cell cycle arrest was activated in these cell lines by a significant increase in the levels of cyclin D1 (*p* < 0.001). Furthermore, the reduction of apoptosis suppression by increasing the levels of caspase 3 was remarkable in HepG2 and MCF-7. For MDA-MB-231, the levels of p53 significantly increased in the cells treated with the plant extract, whereas no significant changes were produced in the levels of cyclin D1 and caspase 3.

### Network pharmacology

#### Screening of candidate ingredients

**Fig 8:**
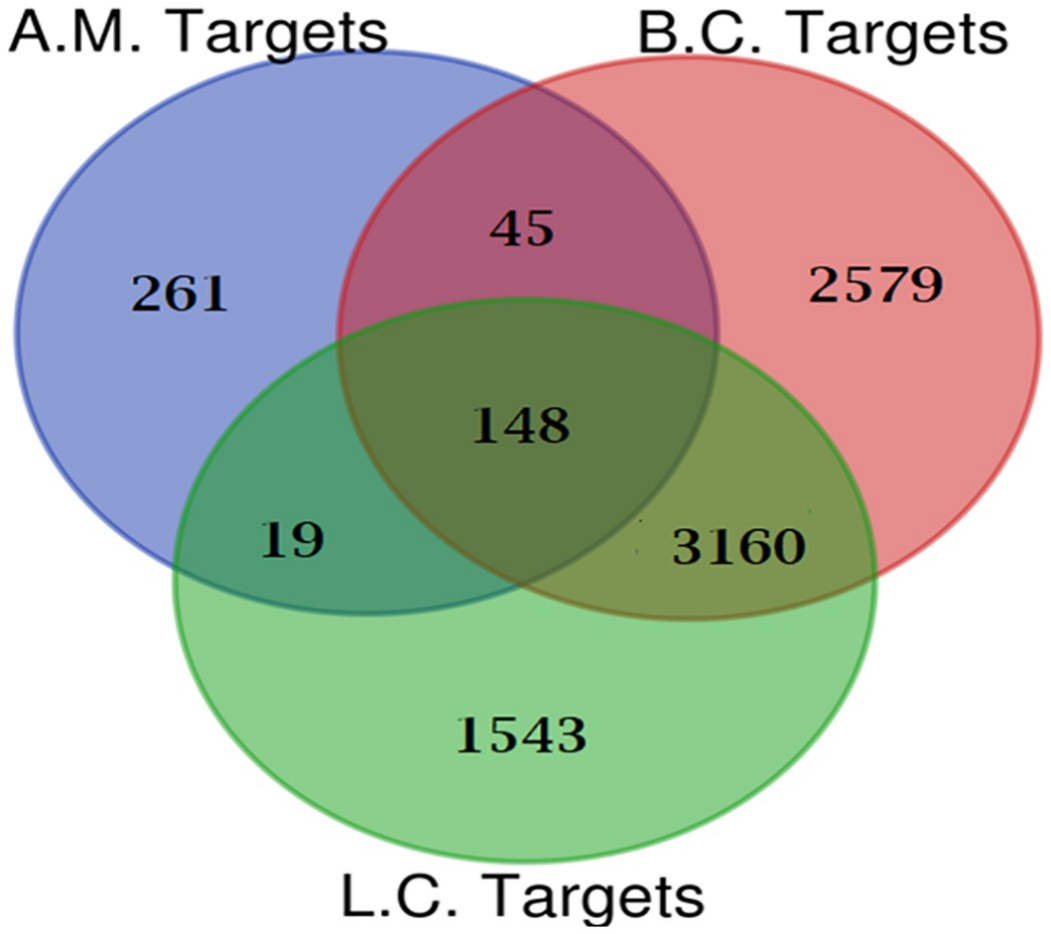
Venn diagram of active metabolites (AM) targets and disease targets [breast cancer (BC) and liver cancer (LC)].

Five active metabolites present in *O*. *ficus-indica* (L.) Mill. were identified using SwissTarget, the BindingDB server, and ADMET Lab 2.0. Following these screening procedures, a total of four metabolites and 261 targets were identified. Upon integration with the targets associated with breast cancer (2579) and liver cancer (1543), sourced from the DisGeNET database using FunRich 3.1.3 software’s Venn diagram intersection, 148 common targets were identified, depicted in Fig 8. Ultimately, four metabolites from *O*. *ficus-indica* (L.) Mill. were found to be linked with these shared targets as active constituents.

#### Protein-protein interaction (PPI) network construction and analysis

**Fig 9:**
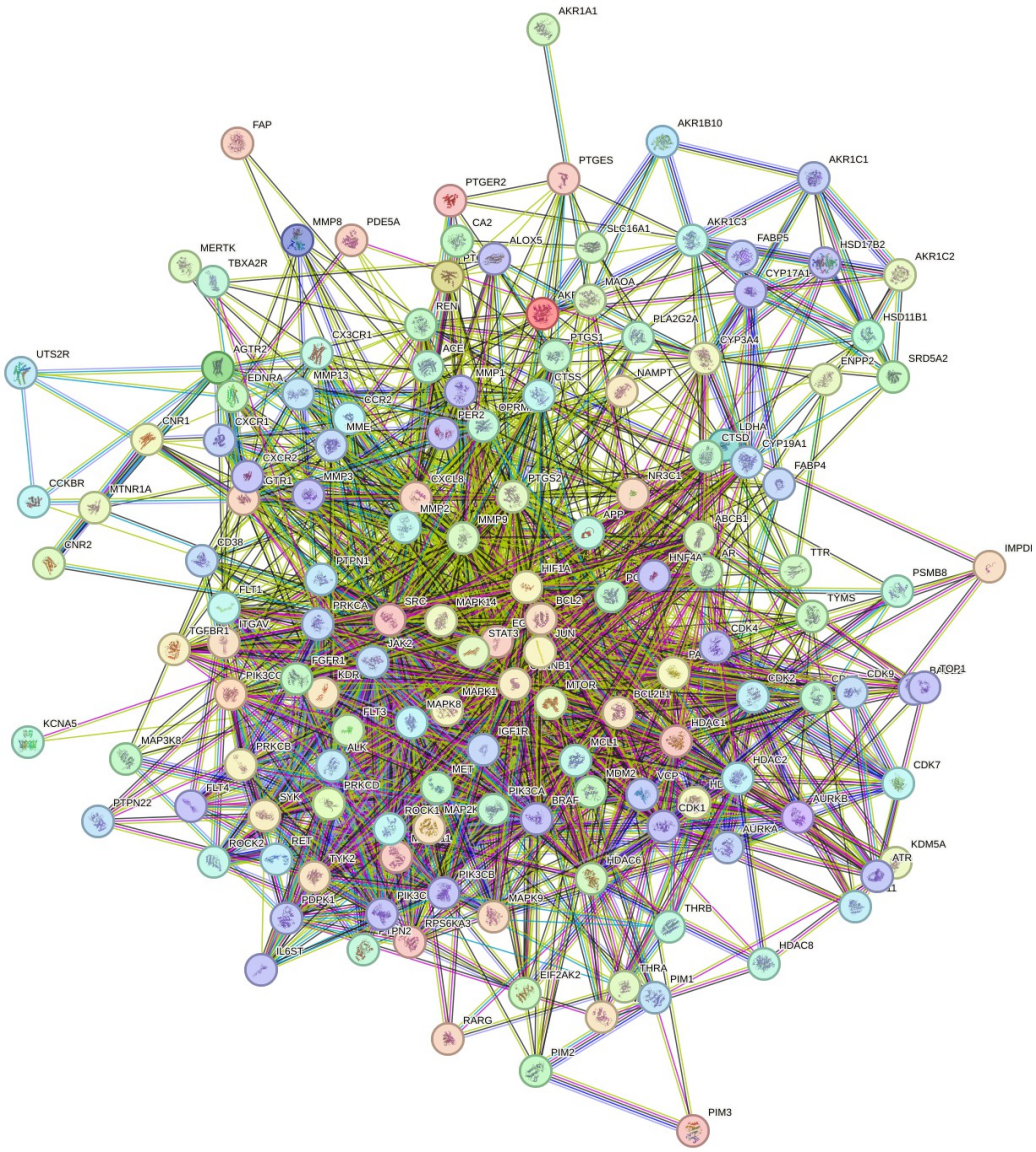
PPI network of *O*. *ficus-indica* (L.) Mill. used as potential targets for breast and liver cancers.

**Fig 10:**
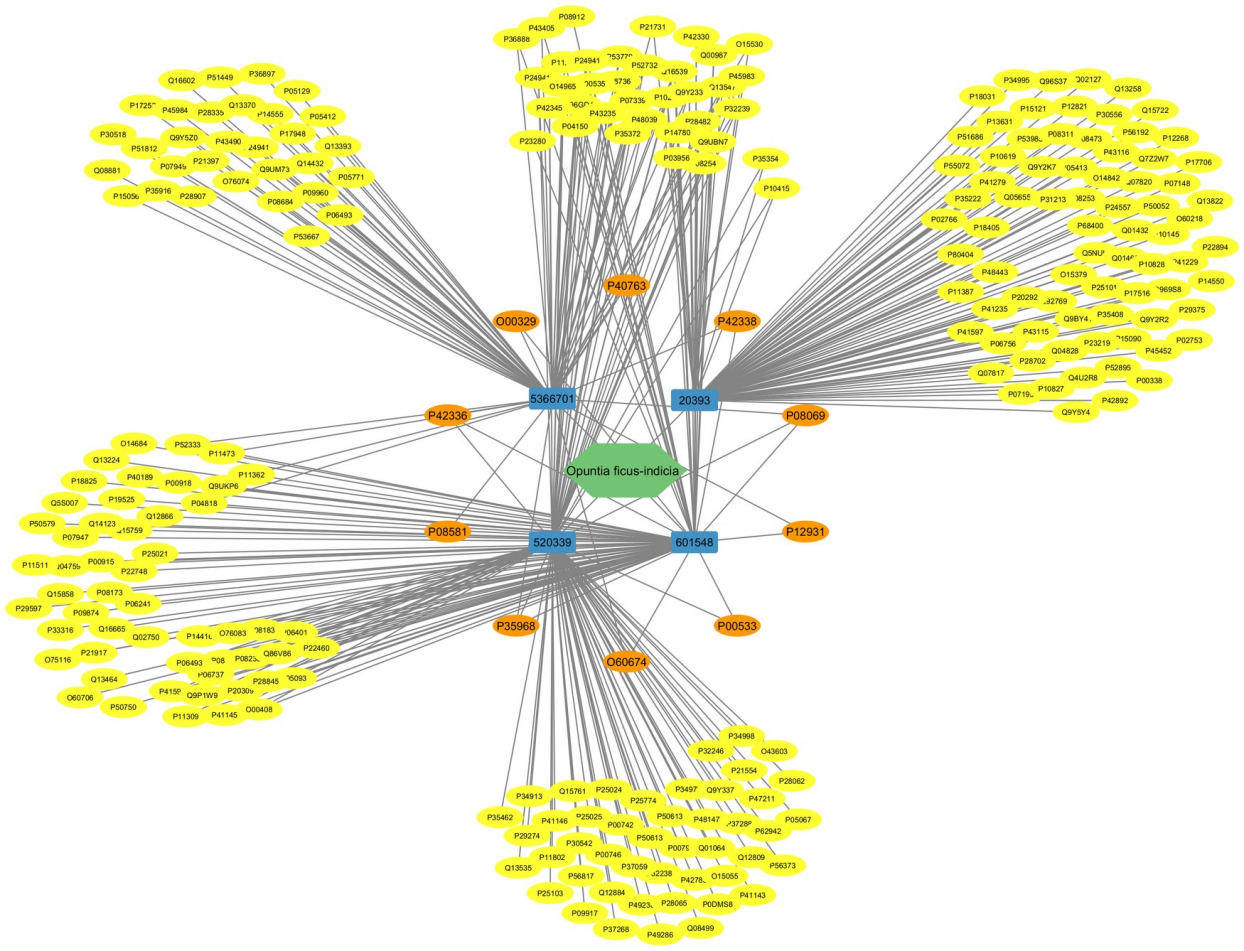
*O*. *ficus-indica* (L.) Mill. metabolites target network. The green polygon is for *O*. *ficus-indica*, the blue rectangles are for metabolites, the orange circles are for the top ten ranked targets by the PPI network, and the yellow circles are for the rest.

Selecting the species "human" from the String database resulted in a network graph with 145 nodes and 1674 edges (Fig 9). Using the TSV file produced from this website and the cytoHubba plug-in Cytoscape, the top ten genes are SRC, PIK3CA, PIK3CB, PIK3CD, JAK2, EGFR, IGF1R, MET, KDR, and STAT3 (Fig 10 and S5 Table).

Three of these genes, PIK3CA, PIK3CB, and PIK3CD, are anticancer-related targets of *O*. *ficus-indica* (L.) Mill. These genes interconnect, interact, and collaborate to suppress the development of breast and liver tumors (Fig 10). This suggested that *O*. *ficus-indica* (L.) Mill. has an anticancer role against breast or liver cancer since the network regulates the key genes.

### GO and KEGG pathway analysis of candidate targets

**Fig 11:**
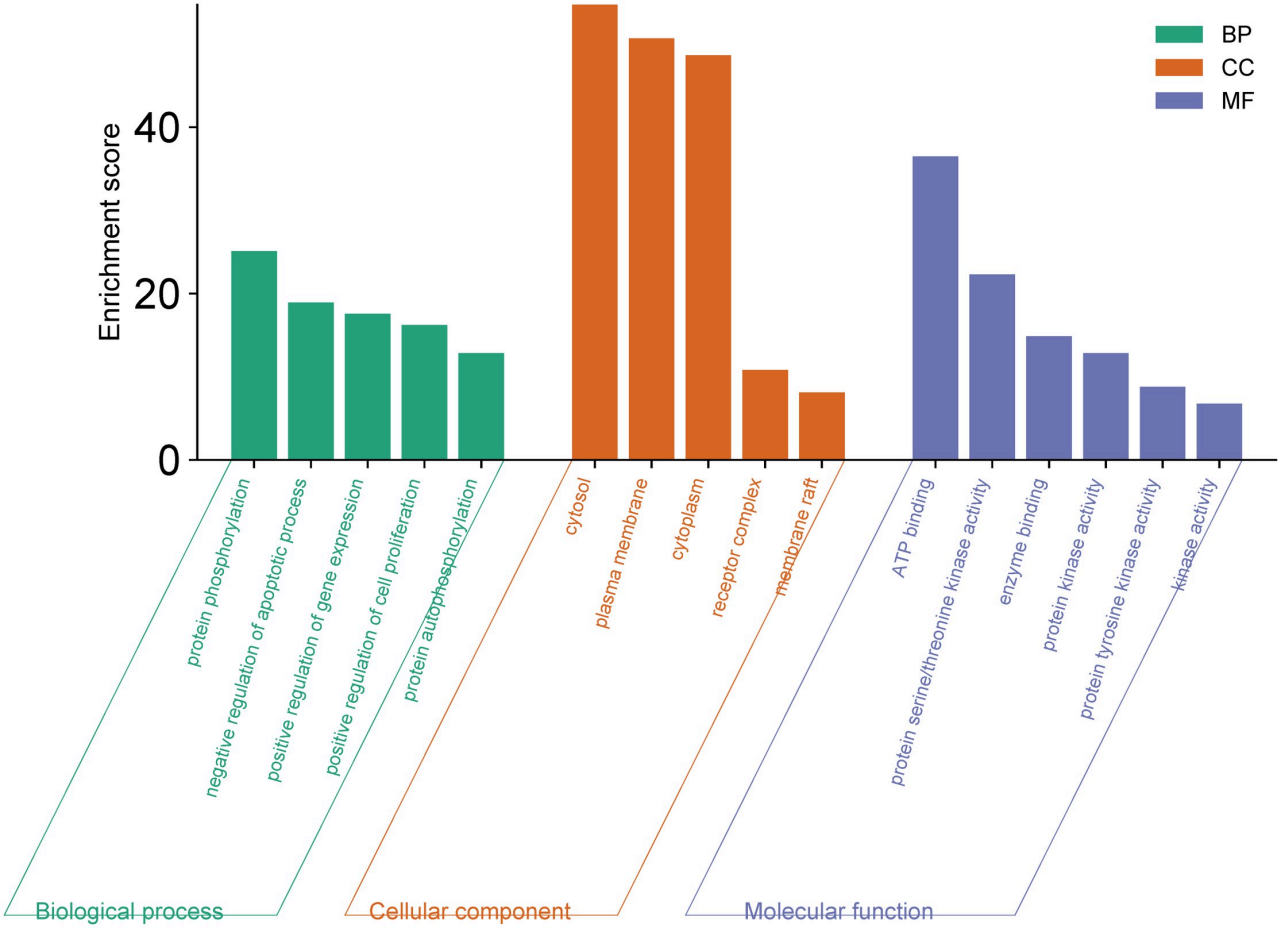
GO enrichment analysis of results for cancer treatment of *O*. *ficus-indica* (L.) Mill.

Enrichment analysis of the 148 identified candidate targets was conducted using Gene Ontology (GO) and Kyoto Encyclopedia of Genes and Genomes (KEGG) through Funrich and ShinyGO 0.77. The analysis encompassed three main aspects: cellular components, molecular functions, and biological processes. Selection criteria for each aspect were based on a significance threshold of *p* < 0.05. Noteworthy cellular components, molecular functions, and biological processes were singled out based on higher enrichment scores (>5), depicted in Fig 11 and S6 Table.

**Fig 12:**
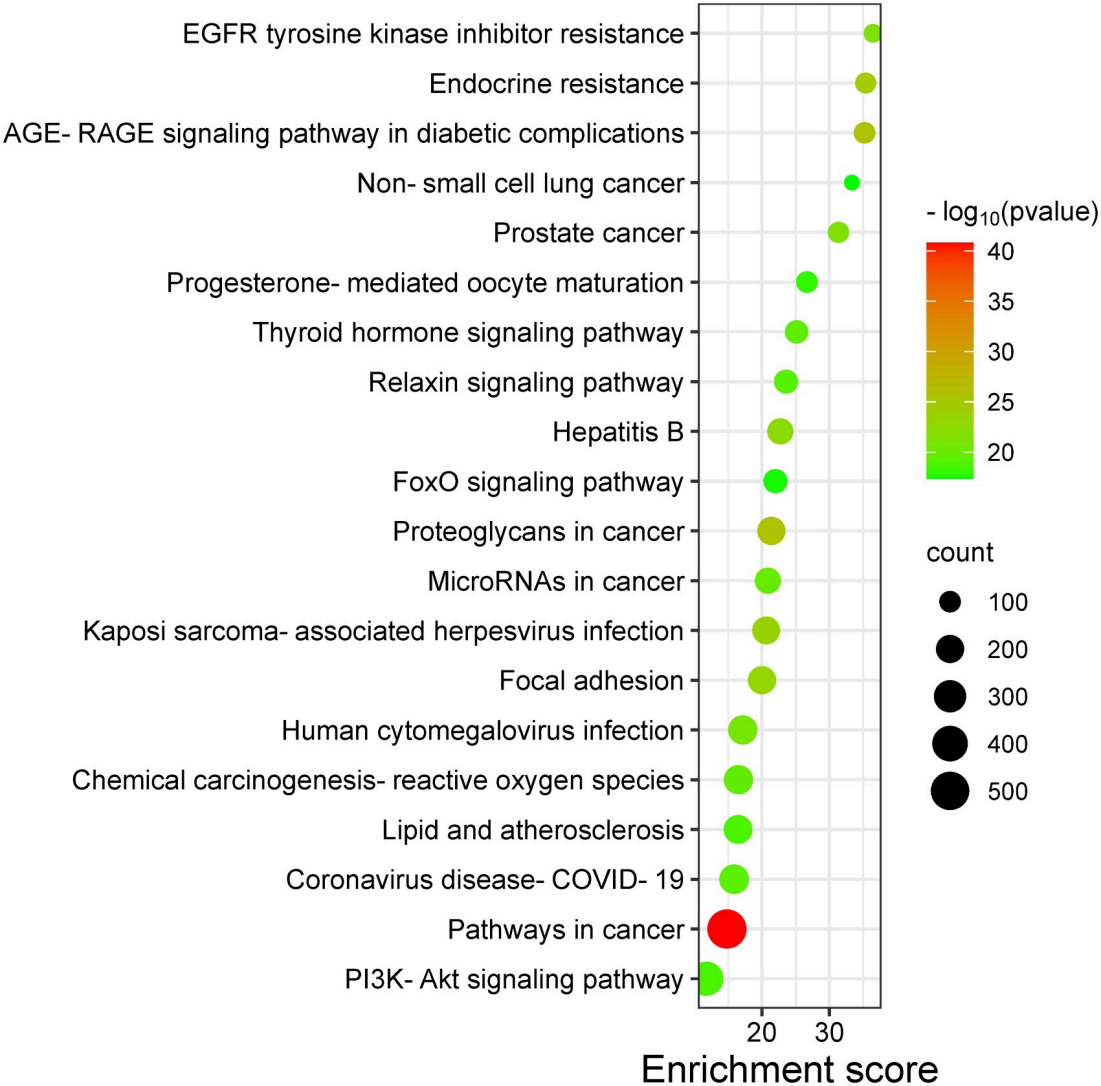
KEGG pathway enrichment analysis of results for cancer treatment of *O*. *ficus-indica* (L.) Mill.

The histogram representation revealed the predominant involvement of candidate targets in processes such as protein phosphorylation, negative regulation of apoptosis, and up-regulation of gene expression, among others. Molecular functions exhibited participation in ATP binding, protein serine/threonine kinase activity, and enzyme binding, among others. Cellular components were predominantly associated with the cytosol, plasma membrane, and cytoplasm. The top 20 pathways, significant at *P* < 0.05, were visualized through a bubble diagram (Fig 12 and S7 Table), highlighting several cancer-related signaling pathways. Notably, the PI3K-Akt signaling pathway emerged as the most significant (Fig 12).

**Fig 13:**
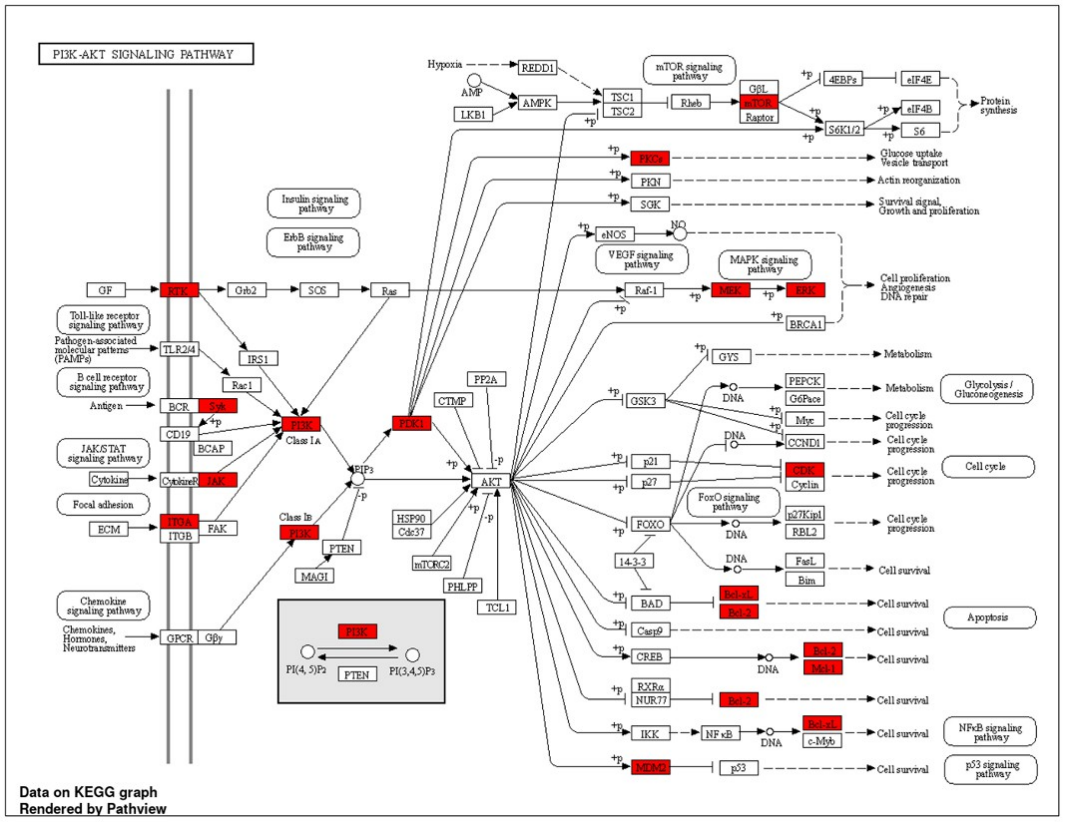
PI3K-Akt pathway.

Further examination of the pathway (Fig 13), sourced from KEGG, elucidated its involvement in regulating protein synthesis, glycolysis, gluconeogenesis, cell cycle, and apoptosis. Additionally, it illustrated synergistic regulation and crosstalk between the NF-ĸB and p53 pathways. These findings suggest a potential antitumor role of *O*. *ficus-indica* (L.) Mill. against breast and liver cancer by modulating the PI3K-Akt signaling pathway [63–65].

### Molecular docking

**Fig 14:**
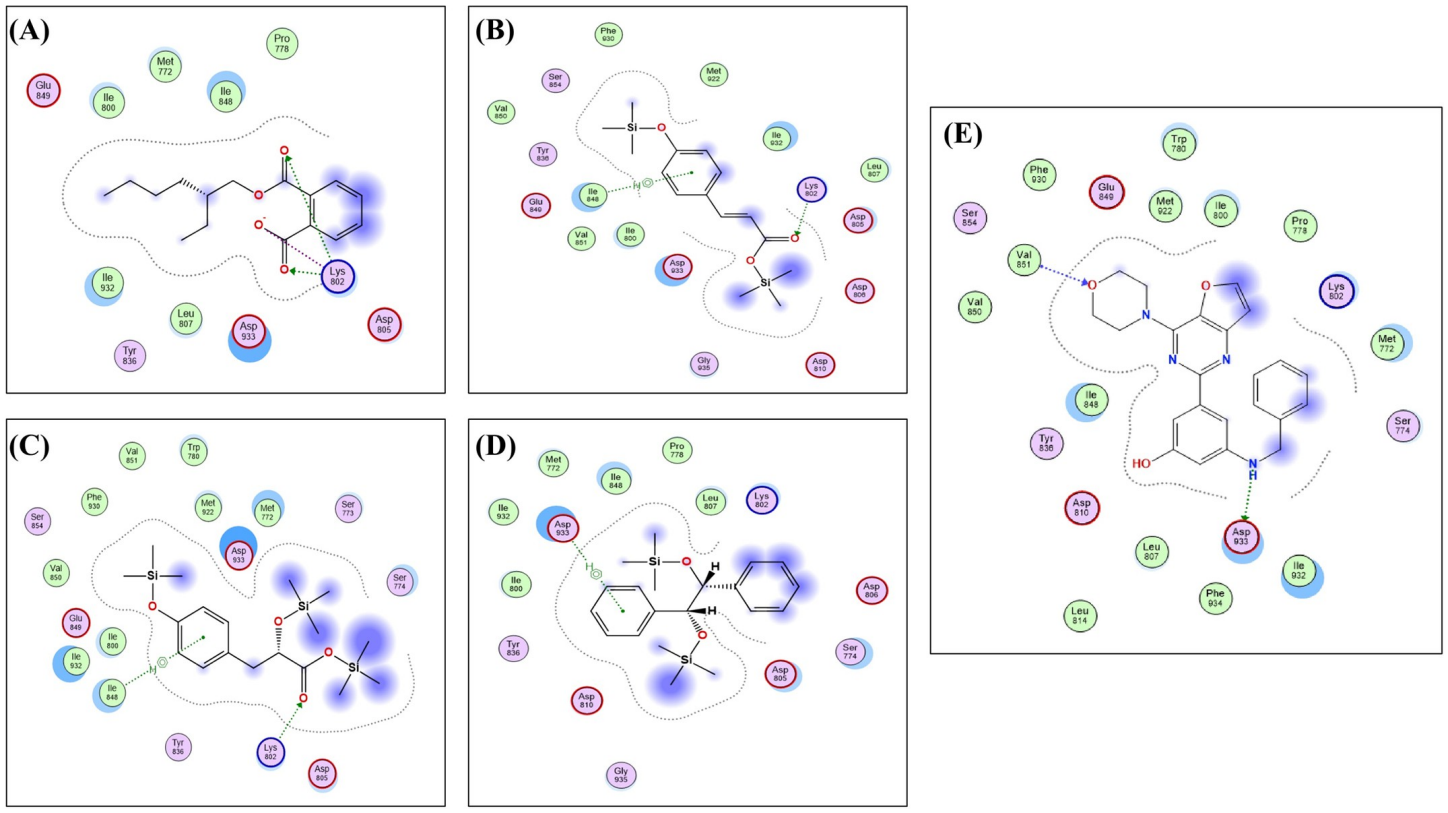
The 2D binding of the active metabolites with a standard PI3K enzyme inhibitor. A) (A) CID: 20393, (B) CID: 5366701, (C) CID: 520339, (D) CID: 601548, and (E) 2D binding interactions of the co-crystallized ligand of PI3K.

To further elucidate the active constituents and their potential targets and mechanisms in treating either breast or liver cancer sourced from *O*. *ficus-indica* (L.) Mill., the most prominent core target, PI3K, identified through an analysis of the protein-protein interaction network, KEGG pathways, and GO enrichment, was chosen for a virtual screening docking simulation study against four active metabolites of *O*. *ficus-indica* (L.) Mill. using Molecular Operating Environment (MOE) software. The findings revealed interactions between all four active metabolites and the core target. Notably, the metabolite with the highest binding affinity to PI3K (CID: 20393) exhibited a binding score of -6.6382 kcal/mol, forming two hydrogen bonds and one ionic interaction with the amino acid residue Lys802 (Table 3 and Fig 14). Compounds (CID: 5366701) and (CID: 520339) demonstrated moderate binding scores of -6.5918 and -6.5911 kcal/mol, respectively, with binding modes involving hydrogen bonding and hydrophobic interactions with Lys802 and Ile848 residues. Conversely, metabolite (CID: 601548) exhibited the weakest binding affinity among the series, with a binding score of -5.2800 kcal/mol, characterized by only one weak hydrophobic interaction with the Asp933 residue (Table 3).

**Table 3:** Docking simulation results of active metabolites against the PI3K enzyme.

Active metabolites PubChem ID (CID)	Binding Score kcal/mol	Key amino acid residues	Type of binding
**20393**	-6.6382	Lys802	2 H-Bonds, Ionic
**5366701**	-6.5918	lle848	Hydrophobic
		Lys802	H-Bond
**520339**	-6.5911	lle848	Hydrophobic
		Lys802	H-Bond
**601548**	-5.2800	Asp933	Hydrophobic

## Discussion

Cancer, including breast cancer, ranks among the leading causes of mortality worldwide, particularly affecting women and contributing to premature death [25, 66, 67]. Despite its widespread use, chemotherapy, the primary cancer treatment, is associated with well-documented side effects [68]. Consequently, there is a concerted effort to develop novel therapeutic strategies utilizing alternative compounds to improve treatment outcomes [69]. Natural products, akin to chemotherapy, are pivotal in both cancer prevention and treatment [70], owing to the diverse array of phytochemicals present in plants that exhibit potent anticancer properties.

The GC-MS analysis of *O*. *ficus-indica* (L.) Mill. flower extract led to 25 compounds. The highest percentages of methanolic constituents were occupied by 1,2,3-tris-(trimethylsilanyloxy)-propane (42.5%), followed by D-fructose and 1,3,4,5,6-pentakis-O-(trimethylsilyl)- (13.2%). Organosilicon compounds represent 57.5% of the total compounds, followed by fatty acid derivatives (17.5%) and monosaccharides and carbohydrates (16.3%). Following these findings, 35 compounds were detected in the GC-MS analysis of the leaves and stem methanolic extracts of *O*. *ficus-indica* (L.) Mill. with a similar classification of polysaccharides and fatty acids [71]. Another study showed that the seed oil of *O*. *ficus-indica* (L.) Mill. had antibacterial activity against *S*. *cerevisiae* and *E*. *coli*, anticancer activity against the PC-3 cells (prostate carcinoma) and the A2780 cell line (ovarian carcinoma), and antiviral activity against the herpes simplex type (2HSV-2) virus [47]. However, malic, quinic, and aconitic acids are known as the most abundant organic acids in the peels and cladodes of *O*. *ficus-indica* (L.) Mill.; they were not detected in the flowers or seeds [37]. On the other hand, gallic acid and caffeic acid derivatives were detected in the flower extracts [37], which are known for their antimicrobial [72] and anticancer properties [73, 74].

Some of the newly discovered compounds had previously been studied for their anticancer activities. Previous studies showed that cinnamic acid derivatives had potential antibacterial [75], autophagy [76], anthelmintic [77], anti-inflammatory, and anti-tyrosinase activities [78], besides their sustainability to reduce the effects of carcinogenic chemicals such as furan and α-dicarbonyl compounds [79]. A previous study showed that gluconic acid derivatives might affect the tumor prognosis by reducing the pH, which increases the acidity of the tumor microenvironment [80]. Poly- and mono-saccharides of glucuronic acid, xylose, galactose, mannose, arabinose, or ribose were considered anticancer polysaccharides because of their immunomodulatory activity, safety usage, and few side effects [81]. Another study showed that the propanoic acid 2,3-bis[(trimethylsilyl)oxy]-, trimethylsilyl ester might be responsible for the antiangiogenic potential and anticancer activities of the *Rumex vesicarius* stem extract [82].

Numerous investigations have been conducted on the cactus pear fruit owing to its methanolic compounds’ antioxidant properties, which are crucial for safeguarding human health against degenerative ailments such as cancer, diabetes, hyperglycemia, hypercholesterolemia, arteriosclerosis, and gastrointestinal issues [83–85]. Studies on human Jurkat T-cell strains have demonstrated that the poly-methanolic compounds derived from *O*. *ficus-indica* (L.) Mill. induce hyperpolarization of the plasma membrane and augment the intracellular calcium pool [86]. In this study, we assessed the anticancer potential of *O*. *ficus-indica* (L.) Mill. against various cell lines, with IC_50_ values of 5 μg/mL for MCF-7, 10 μg/mL for MDA-MB-232, and 80 μg/mL for HEPG2, respectively. Previous research has indicated that the alkaloid extract from *Opuntia* species exhibits significant cytotoxic effects on MCF-7 cells while displaying minimal cytotoxicity towards normal cell lines [87]. Another study indicated that the methanolic extract of *O*. *ficus-indica* (L.) Mill. peel had significant anticancer activity against MDA-MB-231 and HepG2 cancer cell lines with IC_50_ values of 2.00 ± 0.19 and 3.85 ± 0.24, respectively [88]. Additionally, another study highlighted the impact of *O*. *ficus-indica* (L.) Mill. fruit methanolic extract and its betalain pigment, indicaxanthin, on the proliferation of the human colon cancer cell line Caco2 [34].

The emergence of antimicrobial resistance, one of the major drawbacks of the intensive use of antimicrobial agents, including antibiotics and antifungals, necessitates the search for natural compounds with antimicrobial activity to overcome the harmful effects of these microorganisms. For these reasons, in this study, the methanolic extract of the flowers of a famous medicinal plant, *O*. *ficus-indica* (L.) Mill., was tested against different microorganisms, including gram-positive bacteria (*S*. *aureus*), gram-negative bacteria (*E*. *coli* and *P*. *aeruginosa*), and fungi (*C*. *albicans*, *A*. *brasiliensis*, and *S*. *cerevisiae*). This study revealed that this extract provided a promising antimicrobial property for all tested organisms. Our findings were consistent with previous studies that illustrated the antimicrobial potency of *O*. *ficus-indica* (L.) Mill. extracts in a variety of solvents against different bacterial and fungal strains such as *S*. *aureus*, *Bacillus subtilis*, *P*. *aeruginosa*, *Salmonella typhi*, *Klebsiella pneumonia*, and *E*. *coli* [89–92]. It was reported in a recent study that *O*. *ficus indica* (L.) Mill. extracts can inhibit the growth of *S*. *aureus*, *Salmonella typhi*, *Helicobacter pylori*, and *E*. *coli*, with the highest antibacterial activity against *S*. *aureus* and low activity seen against *Salmonella typhi* [90]. Another study showed that the ethanolic extract of the *O*. *ficus indica* (L.) Mill. has excellent antimicrobial activity against *E*. *coli* isolated from patients with UTI [93].

Our investigation underscores the antifungal efficacy of the *O*. *ficus indica* (L.) Mill. extract, particularly notable against *A*. *brasiliensi*. Similarly, preceding research has documented robust antimicrobial attributes of *O*. *ficus-indica* (L.) Mill. oil against yeasts (*Candida parapsilosis* and *Candida sake*) and fungi (*Aspergillus niger*, *Penicillium digitatum*, and *Fusarium oxysporum*) [94]. Another study delineated the antimicrobial potency of *O*. *ficus-indica* (L.) Mill. oil against *S*. *aureus*, *B*. *subtilis*, *E*. *coli*, *Klebsiella pneumoniae*, *S*. *cerevisiae*, and *Penicillium digitatu*, albeit with *Aspergillus niger* displaying the highest resistance to *O*. *ficus-indica* (L.) Mill. oil among other species [47]. Researchers attribute the antimicrobial attributes of *O*. *ficus-indica* (L.) Mill. to the presence of phytochemicals in the tested plant extracts, including methanolic, flavonoids, and carotenoids. Nonetheless, variations in the chemical composition of these phytochemicals, diverse mechanisms of action of their bioactive constituents, varied concentrations, potential interactions with other components, and even the extraction method contribute to differences in antimicrobial activity [95, 96].

In our study, the methanolic extract of *O*. *ficus-indica* (L.) Mill. flowers exhibited robust antioxidant activity, likely attributed to the presence of methanolic compounds, tocopherols, and sterols. Similar observations have been made in previous investigations regarding the free radical scavenging activities of *O*. *ficus-indica* (L.) Mill. seed oil [47, 97], fruit [98], and flowers [21]. Additionally, Lu et al. (2019) demonstrated that the pectin polysaccharides and antioxidant activity of the flower extract augment the medicinal utility of *O*. *ficus-indica* (L.) Mill. in traditional Chinese medicine [99].

The methanolic extract of *O*. *ficus-indica* (L.) Mill. flowers demonstrated promising anticancer effects, eliciting a potential proapoptotic response and inducing cell cycle arrest in MCF-7, MDA-MB-231, and HepG2 cells. Furthermore, treatment with the extract resulted in prooxidant activity in MCF-7 and HepG2 cells, characterized by increased oxidative stress and ROS production. Concurrently, expression levels of p53, caspase 3, and cyclin D1 were upregulated in MCF-7 and HepG2 cells. Consistent with our findings, prior studies have shown that isorhamnetin glycosides or cladode flour extracts of *O*. *ficus-indica* (L.) Mill. induce cytotoxic activities against colon cancer cell lines HT29 and Caco-2 via apoptosis modulation through the caspase cascade [35]. Another study indicated a dose-dependent apoptotic effect of the methanolic fruit extract of *O*. *ficus-indica* (L.) Mill. against Caco-2 cells by upregulating the tumor suppressor gene p16INK4a [34]. Similarly, the chronic myeloid leukemia cell line K562 underwent apoptotic behavior following treatment with betanin derived from *O*. *ficus-indica* (L.) Mill. fruits [100]. Additionally, alkaline fractions of the fruit extract induced cytotoxic activities against HT-29 and Caco2 cells, accompanied by an increase in caspase 3 levels [35]. Moreover, the methanolic extract of the fruit enhanced ROS levels in ovarian cancer cells (OVCA420, SKOV3), demonstrating significant proapoptotic properties mediated through the NF-κB/p-SAPK/JNK/p-AKT signaling pathway [36]. Kim et al. (2015) demonstrated significant cytotoxicity of different extracts of *O*. *ficus-indica* (L.) Mill. stems against SW480 colon and MCF7 breast cancer cells, evidenced by reduced COX-2 expression levels and an elevated Bax/Bcl2 ratio indicative of apoptosis [101].

Moreover, the utilization of network pharmacology and the PPI network approach elucidated the significance of three pivotal genes (PIK3CA, PIK3CB, and PIK3CD) as potential anticancer targets associated with *O*. *ficus-indica* (L.) Mill. These genes form the central axis of the PI3K-Akt signaling pathway, primarily regulating processes such as protein synthesis, glycolysis, gluconeogenesis, metabolism, cell cycle, and apoptosis. Additionally, this pathway facilitates synergistic and regulated cross-talk between the NF-ĸB and p53 pathways. These findings align with biological assay studies, demonstrating *O*. *ficus-indica* (L.) Mill. potential proapoptotic effects and ability to induce cell cycle arrest in MCF-7, MDA-MB-231, and HepG2 cells, suggest a significant role for *O*. *ficus-indica* (L.) Mill. in treating breast and liver cancers by modulating the PI3K-Akt signaling pathway. Molecular docking simulations further validated the impact of *O*. *ficus-indica* (L.) Mill. active ingredients on their presumed target, PI3K, as a potential mechanism for breast or liver cancer treatment. Remarkably, the highly bound active metabolite (CID: 20393) was successfully bound to PI3K, specifically to the key amino acid residue Lys802, validating its potential inhibitory effect on the target enzyme and supporting its anticancer properties [102]. These studies, coupled with our findings, underscore the potential cytotoxicity and anticancer activities of *O*. *ficus-indica* (L.) Mill.

This study shows that *O*. *ficus-indica* (L.) Mill. flower extract has promising antioxidant, antibacterial, and anticancer effects, however, there are still significant limitations. While the *in vitro* results are promising, the bioavailability, metabolism, and toxicity of the substances *in vivo* are unclear, necessitating additional research and clinical trials to establish their safety and efficacy in humans. Furthermore, differences in plant growth circumstances and extraction processes can affect chemical composition, resulting in inconsistent results. Standardization of these processes is required to ensure reproducibility. Molecular docking suggests potential anticancer pathways, particularly via the PI3K-Akt pathway, although these hypotheses require experimental validation. The extract’s selectivity for cancer cells over normal cells was not adequately investigated, raising worries about potential adverse effects. The study also focused on a small number of microbes and cancer cell lines; therefore, expanding the research to include other pathogens and cancer types, as well as investigating combinations with existing treatments, would provide a more complete knowledge of the extract’s therapeutic potential. Overall, the results are encouraging, but more research is needed to confirm the clinical significance and safety of *O*. *ficus-indica* (L.) Mill. flowers.

## Conclusion

Owing to the phytochemical constituents play an important role in biological activities, *O*. *ficus-indica* (L.) Mill. flower extract has many bioactive compounds that reveal significant antibacterial and antifungal activities against tested microorganisms. Thus, the current study emphasized that *O*. *ficus-indica* (L.) Mill. flowers act as natural sources and could be used as alternative eco-friendly antimicrobial agents to control clinical bacterial and fungal pathogens. Moreover, analysis of the phytochemical profiles obtained from the methanolic extract of *O*. *ficus-indica* (L.) Mill. suggested potential therapeutic effects against breast and liver cancer. This effect may be mediated by a group of key genes, including SRC, PIK3CA, PIK3CB, PIK3CD, JAK2, EGFR, IGF1R, MET, KDR, and STAT3, through various pathways such as the PI3K-Akt pathway. These findings were elucidated using combined network pharmacology and molecular docking methods. Further investigations are warranted to validate the mechanistic pathways through which *O*. *ficus-indica* (L.) Mill. exhibits its biological activities *in vivo*.

## Supporting information

S1 Table The active metabolites of *O*. *ficus-indica* and their drug-likeness and oral bioavailability using ADMET Lab 2.0. (XLSX)

S2 Table The prediction of the biological targets by the binding DB server, the Swiss target prediction web tool, and the UniProt database. (XLSX)

S3 Table Results of the DisGeNET database for cancer-associated genes of *O*. *ficus-indica* using the key terms breast cancer and liver cancer. (XLSX)

S4 Table Top ten in network string interactions short (2) ranked by MCC method. (XLSX)

S5 Table GO enrichment analysis of results for cancer treatment of *O*. *ficus-indica*. (XLSX)

S6 Table KEGG pathway enrichment analysis of results for breast and liver cancer treatment of *O*. *ficus-indica*. (XLSX)

S7 Table KEGG pathway enrichment analysis of results for breast and liver cancer treatment of *O*. *ficus-indica*. (XLSX)

S1 Raw image (TIFF)

S2 Raw image (TIFF)

S3 Raw image (TIFF)

S4 Raw image (TIFF)
