# Supplementary figures and images for: Versatile properties of Opuntia ficus-indica (L.) Mill. flowers: In vitro exploration of antioxidant, antimicrobial, and anticancer activities, network pharmacology analysis, and In-silico molecular docking simulation
Source: PLoS One. 2024 Nov 4;19(11):e0313064. doi: 10.1371/journal.pone.0313064 (PMC11534206; doi:10.1371/journal.pone.0313064)

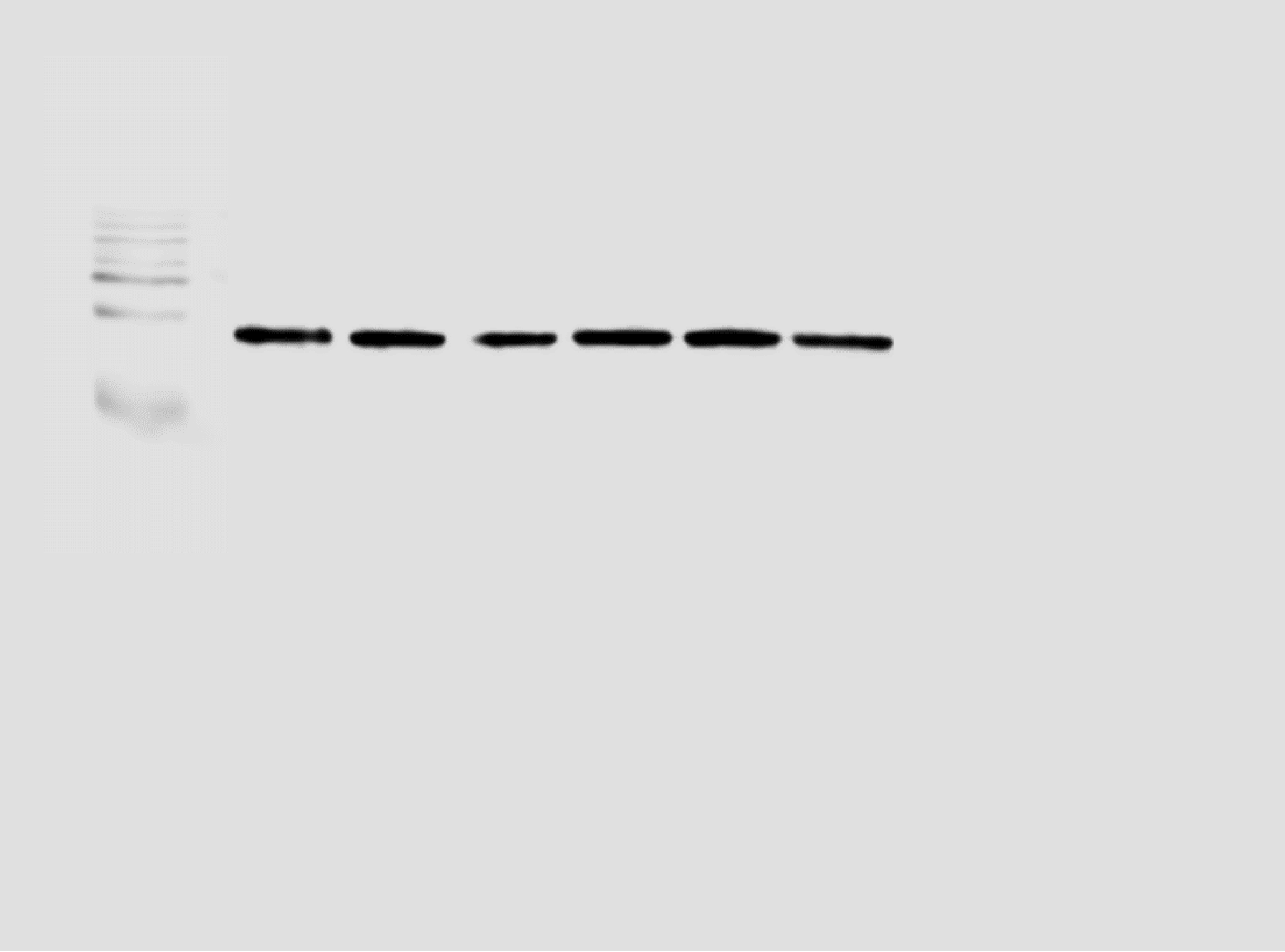

Supplement: S1 Raw image — (TIFF) [file pone.0313064.s008.tiff]

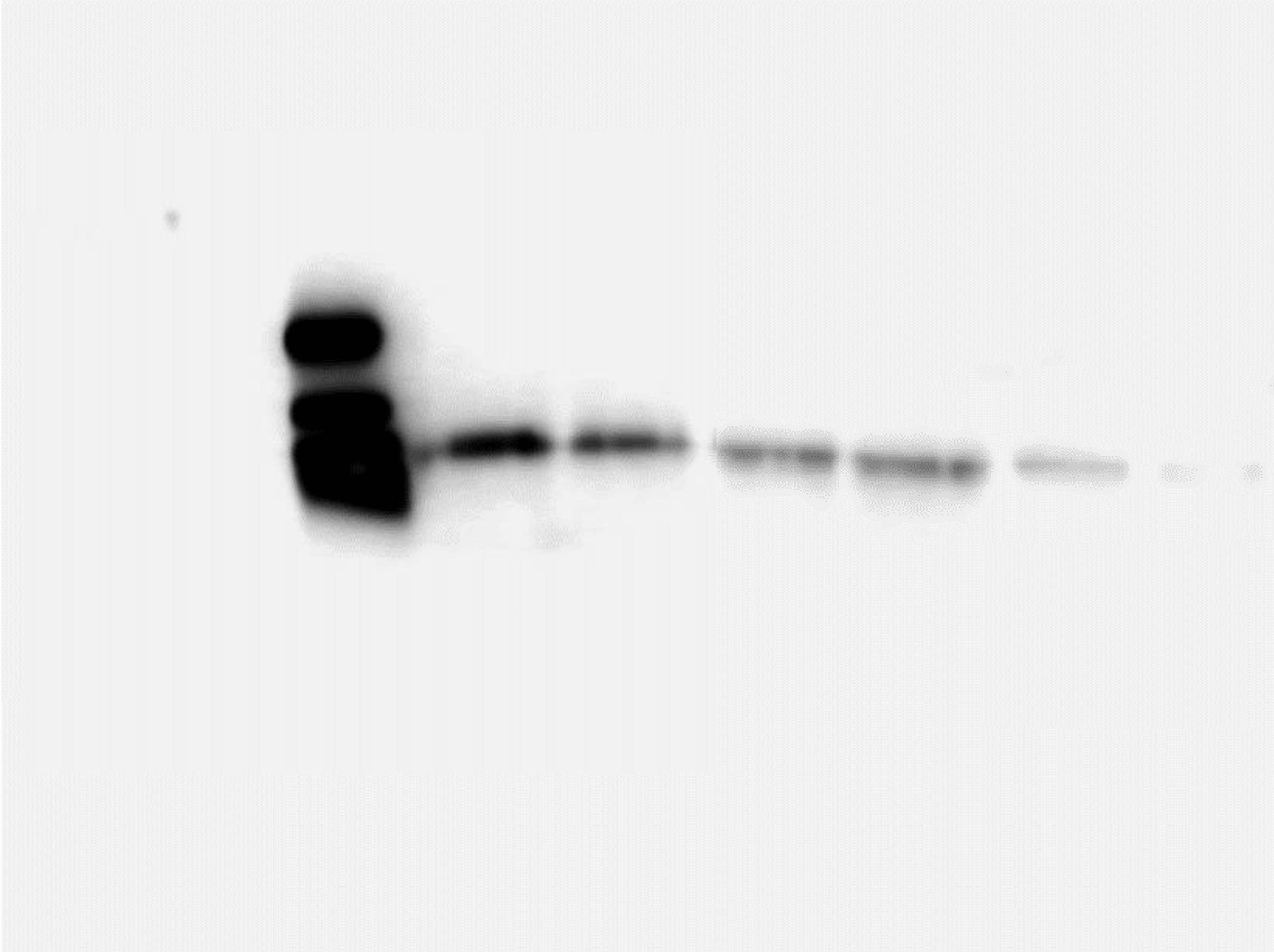

Supplement: S2 Raw image — (TIFF) [file pone.0313064.s009.tiff]

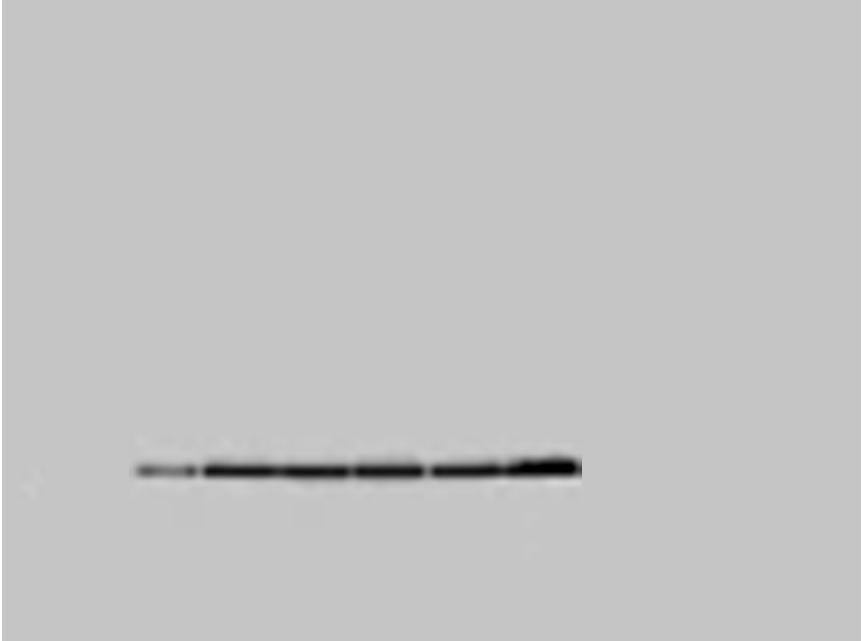

Supplement: S3 Raw image — (TIFF) [file pone.0313064.s010.tiff]

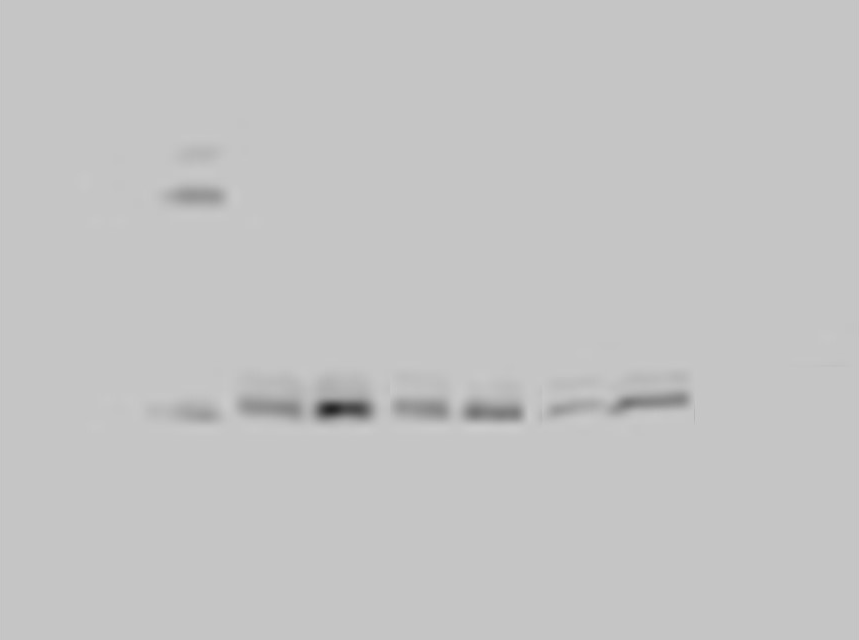

Supplement: S4 Raw image — (TIFF) [file pone.0313064.s011.tiff]
